# MAP3K19 Is Overexpressed in COPD and Is a Central Mediator of Cigarette Smoke-Induced Pulmonary Inflammation and Lower Airway Destruction

**DOI:** 10.1371/journal.pone.0167169

**Published:** 2016-12-09

**Authors:** Stefen A. Boehme, Karin Franz-Bacon, John Ludka, Danielle N. DiTirro, Tai Wei Ly, Kevin B. Bacon

**Affiliations:** 1 Axikin Pharmaceuticals, Inc., San Diego, California, United States of America; 2 DNA Consulting, Inc., San Diego, California, United States of America; University of Kansas Medical Center, UNITED STATES

## Abstract

Chronic obstructive pulmonary disease (COPD) is characterized by persistent airflow limitation and lung inflammation resulting in a progressive decline in lung function whose principle cause is cigarette smoke. MAP3K19 is a novel kinase expressed predominantly by alveolar and interstitial macrophages and bronchial epithelial cells in the lung. We found that MAP3K19 mRNA was overexpressed in a limited sampling of lung tissue from COPD patients, and a closer examination found it to be overexpressed in bronchoalveolar macrophages from COPD patients, as well as the bronchial epithelium and inflammatory cells in the lamina propria. We further found MAP3K19 to be induced in various cell lines upon environmental stress, such as cigarette smoke, oxidative and osmotic stress. Exogenous expression of MAP3K19 in cells caused an upregulation of transcriptionally active NF-κB, and secretion of the chemokines CXCL-8, CCL-20 and CCL-7. Inhibition of MAP3K19 activity by siRNA or small molecular weight inhibitors caused a decrease in cigarette smoke-induced inflammation in various murine models, which included a decrease in pulmonary neutrophilia and KC levels. In a chronic cigarette smoke model, inhibition of MAP3K19 significantly attenuated emphysematous changes in airway parenchyma. Finally, in a viral exacerbation model, mice exposed to cigarette smoke and influenza A virus showed a decrease in pulmonary neutrophilia, pro-inflammatory cytokines and viral load upon inhibition of MAP3K19. Collectively, these results suggest that inhibition of MAP3K19 may represent a novel strategy to target COPD that promises to have a potential therapeutic benefit for patients.

## Introduction

Tobacco smoke exposure is a chief contributor to the rapidly rising worldwide health burden, and the most common smoking-related pulmonary manifestation is chronic obstructive pulmonary disease (COPD) [1]. Approximately 10% of the world’s population is suffering from COPD, and it is currently the third leading cause of death [2, 3]. COPD is characterized by progressive and irreversible airflow limitation [4]. The mechanism underlying COPD is poorly understood, but the disease is thought to have multiple interactive components that cause the chronic obstructive bronchiolitis and emphysema, which are the hallmarks of COPD, and ultimately result in a progressive decline in lung function [5, 6]. The inflammatory arm of COPD is characterized by increased number of neutrophils, alveolar macrophages and T lymphocytes into the pulmonary environment [7–9]. Tissue remodeling is another component of COPD, likely driven by the chronic inflammation, and is manifested by thickening of the airway walls, destruction of the lung parenchyma and enlargement of the airspaces, the loss of lung elasticity and closure of the small airways [10–13]. Patients with COPD often experience acute exacerbations, which are usually triggered by viral or bacterial infections, and become increasingly frequent as disease progresses [14, 15].The main pharmacological therapy for treatment of COPD is long acting bronchodilators, which provide only symptomatic relief, as COPD progression is typically refractory to steroid treatment [13, 16]. As there are currently no treatments that block COPD progression, a greater understanding of the disease process providing new therapeutic approaches is necessary.

The major risk factor for the development and progression of COPD is chronic exposure to cigarette smoke, air pollution or other noxious gases [17–19]. Irritants from tobacco smoke can trigger innate immune mechanisms directly, resulting in NF-κB activation, presumably from toll like receptor (TLR) activation [20]. Alternatively, cigarette smoke also places lung cells under oxidative stress, which in turn can trigger inflammation, protease-anti-protease imbalances and apoptosis [19, 21]. Thus, oxidative stress, or the dysregulation between oxidant and antioxidant levels in a cellular microenvironment, via cigarette smoke, has become recognized as a major predisposing factor of COPD [19, 22].

Cigarette smoke has also been shown to cause cytokine and chemokine secretion, leading to the pulmonary recruitment of neutrophils; the increased numbers of which positively correlate with COPD progression [20, 23]. Typically, neutrophil infiltration is a defense mechanism to remove pathogens and repair injured tissue [24]. However, in COPD, neutrophils migrate into the lungs, particularly in response to CXCL-8 [25, 26], and the activated neutrophils can release reactive oxygen species, creating a positive feedback loop for oxidative stress. Neutrophils can also release factors that attract more neutrophils out of the bloodstream, thereby perpetuating the inflammation further [27]. Finally, activated neutrophils secrete enzymes which may contribute to emphysema by degrading elastin and collagen and stimulating mucous secretion [27].

MAP3K19 is an evolutionarily conserved, novel kinase whose expression in humans and mice was shown to be highest in the lung and trachea, and more specifically, concentrated in the bronchial epithelium, macrophages and neutrophils [28]. It was previously shown in rice plants that MAP3K19 gene expression was increased upon exposure to high salt conditions [29]. Together with our results, this raises the possibility that MAP3K19 is part of an evolutionary conserved pathway involved in a cellular response to various environmental stressors. Our previous investigations have shown that MAP3K19 regulated the nuclear translocation of activated phospho-Smad2/3 following TGF-β stimulation of cells, as inhibition of the kinase blocked the nuclear accumulation of the P-Smads. Furthermore, this resulted in decreased TGF-β-mediated gene transcription and protein production. We have now extended these findings to show that MAP3K19 is upregulated in various cells following oxidative stress. In addition, in the present study we have demonstrated that MAP3K19 was over-expressed in COPD lung tissue. An examination of the role of MAP3K19 in the COPD disease process revealed that inhibition of MAP3K19 by either siRNA or small molecule inhibitors strongly decreased the cigarette smoke-induced pulmonary inflammation in an acute model. Inhibition of MAP3K19 in a chronic model of cigarette smoke exposure resulted in markedly less airway tissue destruction and emphysema-like changes. Finally, decreased inflammation and viral load were observed when MAP3K19 was inhibited in a viral exacerbation model of COPD.

## Materials and Methods

### Materials

**Fig 1:**
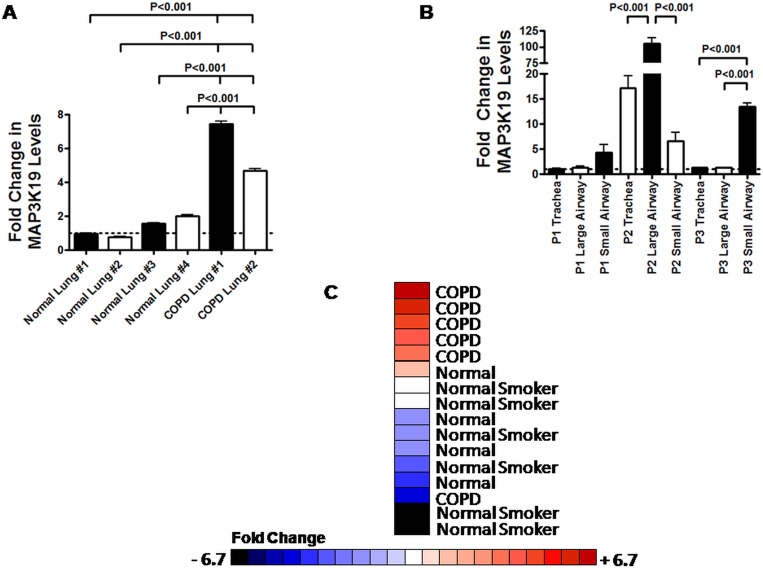
MAP3K19 is overexpressed in COPD lung and bronchoalveolar lavage macrophages from COPD patients. (A) MAP3K19 RNA expression in four normal human lung samples and two COPD lung samples was examined by RT-qPCR and normalized to GAPDH levels, showing higher levels of expression in COPD diseased tissue compared to normal controls. The MAP3K19 expression in the Normal Lung #1 sample was arbitrarily assigned a value of one (denoted by the dotted line) and fold expression of MAP3K19 in all other tissues examined were relative to that sample. The average fold expression is shown ± SEM. (B) MAP3K19 expression was examined in either the trachea, large airways (primary or secondary branches) or small airways (tertiary branches or below) from three COPD patients (P1, P2 & P3) by RT-qPCR analysis. The levels of MAP3K19 was normalized to GAPDH levels, and the expression level of patient 1 trachea was arbitrarily assigned a value of one (denoted by the dotted line). MAP3K19 levels in all other samples were relative to that sample. This experiment was repeated twice, each time in duplicate, and the average fold expression is shown ± SEM. (C) MAP3K19 is over-expressed in the bronchoalveolar lavage macrophages from COPD patients. Heatmap showing MAP3K19 expression in BAL macrophages isolated from Gold Stage I & II COPD patients (n = 6), normal controls (n = 5) and healthy smokers (n = 6) as determined by RT-qPCR analysis. The values are normalized to GAPDH expression levels and the fold changes are depicted in the color scale. The RT-qPCR is representative of two experiments (biological replicates) performed in duplicate. Statistical analysis (ANOVA followed by Tukey-Kramer pair-wise test) of MAP3K19 expression levels between the different patient groups revealed that the difference in the mRNA levels between the COPD group, omitting the lowest expressing patient, and either the normal patients or healthy smokers was significant, P<0.01. Significant differences between groups was determined by ANOVA analysis followed by the Tukey-Kramer pair-wise tests, as detailed in the Materials and Methods. P<0.05 were considered significant. Patient demographics are shown in the S1–S3 Tables.

All reagents were obtained from Sigma-Aldrich Chemical Co. (St. Louis, MO, USA) unless otherwise stated. SB265610 was purchased from Selleckchem (Houston, TX, USA). TGF-β1, TNF-α, IL-4, IL-13 and IFN-ɣ were purchased from R&D Systems (Minneapolis, MN, USA) and reconstituted according to the instructions. The 8-iso-Prostaglandin F2α competitive ELISA was obtained from Cell Biolabs, Inc. (San Diego, CA, USA) and used according to the instructions. Healthy individual and COPD patient lung tissue utilized in Fig 1A was obtained from Analytical Biological Services, Inc. (Wilmington, DE), and healthy human lung RNA was obtained from Clontech Laboratories, Inc. (Mountain View, CA, USA) and Life Technologies (Carlsbad, CA, USA). Cell supernatants or BAL fluid were assayed for CXCL-8, MIP-3α, MCP-3 or KC by ELISA purchased from R&D Systems and used according to their instructions. MAP3K19 inhibitors Compounds A, B, C, D & E are proprietary, highly potent and selective MAP3K19 small molecular weight inhibitors that were developed as part of the Axikin Pharmaceuticals, Inc. portfolio of compounds (US Patent number 60/096308).Inhibition assays examining the activity of these Compounds on the kinase activity of MAP3K19 showed the following IC_50_ values: Compound A –16 nM; Compound B– 5.2 nM; Compound C– 8.7 nM; Compound D –19.6 nM; and Compound E– 200 nM.

### Gene Expression Analysis

RNA was isolated from cells and tissue sections as previously described [28]. The COPD lung tissue analyzed in Fig 1B was ethically obtained from recently deceased patients from a tissue network according to standard procedures (Biopta Ltd., Glasgow, UK). The trachea, large airway (primary or secondary branches), and small airway (tertiary branches and below) were dissected from the lungs and stored in RNAlater (Qiagen). The RNeasy kit (Qiagen, Valencia, CA, USA) was used to isolate RNA, and total RNA was reverse transcribed using SuperScript III (Life Technologies). Fifty nanograms of cDNA was assayed using the 7900HT Fast Real-Time PCR system (Applied Biosystems, Inc., Foster City, CA, USA) using Power SYBR Green PCR Master Mix (Applied Biosystems, Inc.) and performed by the CFAR Genomics and Sequencing Core of UCSD/VMRF (San Diego, CA, USA). Each sample was run in duplicate. The oligonucleotide primers (125 nM final concentration) for GAPDH were designed and validated by Integrated DNA Technologies (San Diego, CA, USA), and the MAP3K19 primers used were: sense primer: 5’ aatggcacccacagtgacatgctt 3’; anti-sense primer: 5’ ccctcggtgtgctccgatgtaaaa 3’. The amplification efficiencies were determined for each target gene and normalized to human GAPDH, and the delta Ct was calculated using the comparative analysis method.

### Histological Analysis

**Fig 2:**
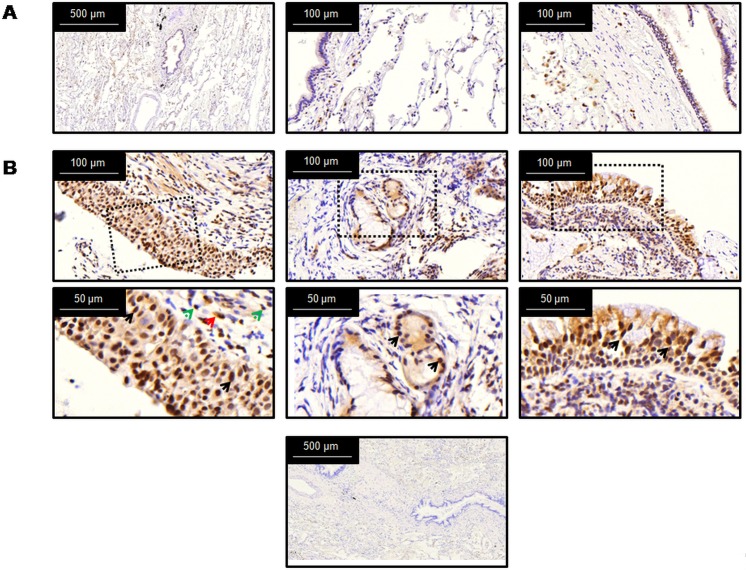
Immunohistochemical analysis showed that MAP3K19 expression appeared greater in COPD lung than normal human lung. (A) Normal human lung was stained with the anti-MAP3K19 polyclonal rabbit antibody, RabK19, shown in brown, and counterstained with hematoxylin. Expression of MAP3K19 is found in the ciliated bronchial epithelial cells, alveolar and interstitial macrophages, neutrophils, and some type II pneumocytes. (B) COPD biopsies are stained with anti-MAP3K19 (RabK19) antibody. In the left panels, the majority of the airway epithelial cells are stained (black arrows), and in the lamina propria the macrophages (red arrow) and lymphocytes (green arrows) are positive for MAP3K19 expression. The middle panels show the submucosal glands staining positive for MAP3K19. The right panels show the airway epithelial cells, particularly the nuclei, positive for MAP3K19 staining (black arrows), and variable staining of the inflammatory cells in the lamina propria. Images are shown from three COPD patients, and are representative of ten patients from whom biopsies were procured (diagnosed with COPD from 1–25 years). The lower panel is the negative control, an isotype control antibody. The patient demographics are listed in the S4 Table.

Antibodies directed against MAP3K19 were produced by Pro-Sci Inc. (Poway, CA, USA) and were used as previously described [28]. Histological analysis was performed by Reveal Biosciences, Inc. (San Diego, CA, USA) and the MAP3K19 rabbit polyclonal antibody, RabK19, was previously validated and titrated by Reveal Biosciences. For the images in Fig 2, normal adult lung sections were obtained from Bio Options (Brea, CA, USA), and the COPD sample biopsies were commercially obtained from Cureline Inc. (South San Francisco, CA, USA). Ten biopsy samples were examined, and the COPD patients ranged from 52–85 years old, smoked 1–2 packs per day for 35–70 years, were all male patients and were diagnosed with COPD for 1–25 years. Nine of the patients were not receiving any therapy at the time of the biopsy and one patient started dexone 4 days prior to the lung biopsy. Signed informed consent was provided by all patients and Cureline’s procurement protocols were approved by local IRB and Ethical Committees and that complied with international and local regulations and guidelines. All sections used including the 10 COPD biopsy samples and the normal lung were processed identically following fixation in 10% neutral buffered formalin. The sections were dewaxed and antigen retrieval was performed using a 1X DIVA Decloaker (BioCare Medical (Concord, CA, USA) for twenty minutes. Non-specific protein binding was blocked with 3% Normal Donkey Serum in PBS + 0.1% Triton X-100 (Sigma-Aldrich Chemical Co.). Slides were stained with the primary antibody, RabK19, at a 1:100 dilution, and were detected by Bond Polymer Refine Detection System (Novacastra Reagents, Leica Biosystems Inc.) with 3’3’-diaminobenzidine (DAB, brown) as the substrate and a hematoxylin nuclear counterstain (blue).The negative control slide was stained with an isotype matched control antibody. The immunohistochemical analysis of was performed on a Leica Bond automated immunostainer (Leica Biosystems, Inc., Buffalo Grove, IL, USA). Slide imaging was performed in bright field using a Pannoramic Scan from 3D Histech (Perkin Elmer, Waltham, MA, USA).

### Isolation of Human Bronchoalveolar Lavage Macrophages

Bronchoalveolar lavage (BAL) from human patients was approved and performed at the Universita degli studi di Roma Tor Vergata under the supervision of Dr. Cesare Saltini as described previously [28]. The protocol for the bronchoalveolar lavage of human subjects and material obtained was part of a collaborative project between ReDD (Research for Drug Discovery) Tor Vergata,the Cattedra Malattie Apparato Respiratorio & Centro Interdipartimentale per lo Studio delle Malattie Rare del Polmone e della Fibrosi Polmonare, Facoltà di Medicina e Chirurgia of the University of Rome, and Axikin Pharmaceuticals, Inc. The patients provided written consent for the study, and the consent procedure and investigational protocol was approved by the Policlinico Universitario (University Hospital) Tor Vergata Ethics Committee (SCS/REDD/08 n. 123/08). BAL macrophages were isolated from four healthy patients, six healthy smokers and six patients with COPD, classified as Gold stage I or II. None of the patients were on immunosuppressive therapy at the time of the study. The cells from the BAL lysates were assayed for viability by trypan blue cell counts and cell purity was determined by flow cytometric analysis or Diff Quick (Thermo Fisher Scientific, Waltham, MA, USA) stained cytocentrifuged slide preparations. BAL samples that had greater than 5% ciliated epithelial cells were discarded. Alveolar macrophages were adhered to plastic tissue culture dishes (Corning, Thermo Fisher) for thirty minutes at 37°C for further purification. RNA was subsequently isolated from the purified BAL macrophages (>95% purity) using the RNA/DNA/Protein Purification kit from Norgen Biotek (Thorold, Ontario, Canada) and used for RT-qPCR as described previously [28].

### Cell Culture

A549 (Cat. # CCL-185), HeLa (Cat. # CCL-2), THP-1 (Cat. # TIB-202), Beas-2B (Cat. # CRL-9609), HEK293 (Cat. # CRL-1573), U937 (Cat. # CRL-1593.2) and NIH3T3 (Cat. # CRL-1658) cells were all purchased from the ATCC (Manassas, VA, USA) and cultured according to ATCC instructions. Briefly, F12-K (A549) and DMEM (HeLa, HEK293, NIH3T3 and THP-1) (both medias from ATCC) base medias were supplemented with 10% FCS (Life Technologies), 2 mM glutamine (Corning Life Sciences, Tewksbury, MA, USA), 10 mM HEPES (Corning Life Sciences), 100 μM non-essential amino acids (Corning Life Sciences), sodium pyruvate (Corning Life Sciences), penicillin and streptomycin (Corning Life Sciences). Beas-2B cells were cultured in BEBM media (Lonza Inc., Allandale, NJ, USA) supplemented with a BEGM kit (Lonza Inc.) without the gentamycin-amphotericin B mix. Cigarette smoke extract (CSE) was made essentially by the method detailed in Eurlings et al. [30]. Filters were removed from 3R4F Research cigarettes (University of Kentucky, Lexington, KY) and CSE was made by bubbling the smoke of one cigarette into 10 mls of full media using a linear pump (2 mL/sec). Cells were stimulated with a 10% CSE solution in the appropriate full media. Cells were transfected with siRNA using the Lipofectamine RNAiMAX Reagent (Life Technologies) directed against MAP3K19 or non-sense siRNA (all of the unmodified siRNAs used in this study were purchased from Qiagen). The sequence of the human MAP3K19 siRNA was 5’ CAUUGGUUGUACUGUGUUUTT 3’, and the non-sense siRNA sequence was 5’ CAUAACGCGUAUACUCGACTT 3’ (Qiagen). The murine MAP3K19 siRNA sequence was 5’ AGAGUGGUUGAGCGAGAUUTT 3’ and the murine non-sense siRNA sequence was 5’ ACUAAGUACGUCGUAUUACTT 3’ (Qiagen). Cells were also transfected with a MAP3K19 expression plasmid (cloned into pcDNA3.1), or a kinase dead mutant of MAP3K19 (K1089R mutation) generated by using the QuikChange II XL Site-Directed Mutagenesis Kit (Agilent Technologies, Santa Clara, CA, USA) and Lipofectamine 2000 (Life Technologies). HEK293 and HeLa cells were also transfected with an NF-κB luciferase reporter plasmid that contains five copies of a NF-κB-binding element that drives transcription of luciferase reporter gene (pGL4.32, Promega Corp., Madison, WI, USA). Luciferase activity was measured using the One Glo Luciferase Assay System (Promega Corp.) according to the manufacturer’s instructions.

### Western Blotting & Protein Analysis

HEK293 cells were treated as described, and nuclear and cytoplasmic extracts were made using the NE-PER Nuclear and Cytoplasmic Extraction Reagents (Pierce, Rockford, IL, USA) in the presence of protease and phosphatase inhibitors (Halt Protease and Phosphatase Inhibitor Cocktail, Pierce). The BCA Protein Assay Kit (Pierce) was used to determine total protein concentration in each sample. Western analysis was essentially performed as previously described [28]. Briefly, twenty micrograms of protein was electrophoresed through 10% polyacrylamide gels (Life Technologies) and transferred to nitrocellulose using the iBlot dry transfer system and reagents (Life Technologies). Blots were incubated for one hour in 10% BSA (fraction V, Sigma-Aldrich) and TBST buffer, and hybridized overnight. All antibodies were purchased from Cell Signaling Technologies (Danvers, MA, USA), except the MAP3K19 rabbit polyclonal antibody RabK19, which was used at 1:250. The Cell Signaling Technologies antibodies were diluted (1:1000) and included anti-phospho-NF-κB p65subunit (Ser 536, Cat. # 3033), anti-NF-κB p50 subunit (Cat. # 3035), and anti-HDAC-1 (Cat. # 2062). SuperSignal West Femto Maximum Sensitivity Substrate (Pierce) was used to develop the Western blots and they were visualized using a Kodak Gel Logic 440 Imaging System (Eastman Kodak, New Haven, CT, USA).

### Animal Models of Cigarette Smoke-Induced Inflammation

#### Acute Challenge Model

Eight–ten week old female BALB/c mice (Charles River Labs, Wilmington, MA, USA) were used for the smoke studies unless otherwise noted. Animals were housed and treated in accordance with guidelines outlined in Animal Care and Use Protocol (Explora Biolabs, San Diego, CA, USA, IACUC Protocol # EB10-006, approved 5/3/2010, unless documented otherwise). The procedure was based on the protocol used by Stebbins et al. [31]. The mice were place in a pexiglass chamber and exposed to the smoke of seven cigarettes each day, each cigarette for a 15 minute period; thus mice got exposed to smoke for 105 minutes per day (3R4F research cigarettes, filters removed, University of Kentucky; or Marlboro^®^ brand cigarettes). A peristaltic pump was used to deliver the smoke to the chamber at a delivery rate of 45 ml/min (Cole Parmer Masterflex, Vernon Hills, IL, USA). The smoke was diluted with medical air prior to reaching the pexiglass chamber. Control mice received only medical air exposure. Mice were exposed to smoke on days one and two, and treated with drugs, vehicle or siRNA on days 1–3. Mice were dosed orally with either Compounds A–E (10 mg/kg unless otherwise noted), the CXCR-2 inhibitor SB265610, or the vehicle (40% Solutol / 10% Ethanol / 50% H_2_O). siRNA was dissolved in sterile PBS (2 mg/ml), and a 50 μl volume was administered intra-tracheally [32]. On day four of the study, approximately 48 hours after the last smoke exposure, mice were sacrificed by isoflurane inhalation and a bronchoalveolar lavage (BAL) was performed by inserting a 20 gauge luer into the exposed trachea and infusing the lungs twice with 0.5 ml of ice cold PBS. Total leukocyte counts were determined, and cytospin slides were prepared for differential cell counts. A minimum of 300 cells were differentiated by standard morphological features. The collected BAL fluid, with the cell pellets removed, were stored at -80°C for ELISA analysis. Following the extraction of BAL fluid, one lobe of lung was inflated with OCT (Thermo Fisher Scientific), snap frozen in a dry ice/ethanol bath, and stored at -80°C until used for histology.

#### Subchronic Challenge Model

The subchronic cigarette smoke exposure model was carried out similarly to the acute model with the major exception being mice were exposed to smoke (1.75 hours per day) on days 1, 2, 5–9, 12–14, and dosed in a therapeutic regimen on days 6–14 with siRNA or PBS intra-tracheally [31]. Bronchoalveolar lavage was performed on day 15 as outlined for the acute model.

#### Chronic Challenge Model

The chronic model was carried out similarly to the acute model except that mice were exposed to cigarette smoke for five days per week for twenty-two weeks. After day 78, the smoke-treated mice were randomized and treated daily for the duration of the study with vehicle, Compound D or E (10 mg/kg, administered orally) or dexamethasone (0.5 mg/kg diluted in PBS, delivered intraperitoneally). On day 156, the mice were sacrificed, weighed, and the lungs were isolated en bloc, with no BAL fluid isolated to preserve the lung structure. Serum was obtained from blood isolated by cardiac puncture, and stored at -80°C until analysis. A metal cannula was secured onto the trachea, and in a fume hood the lungs were carefully perfused with 10% phosphate buffered formalin (PBF) by tracheal instillation at a pressure of 20 cm H_2_O [33]. After inflating the lungs in this manner for 50 minutes, the trachea was tied off and the lungs were removed from the apparatus. The lungs were immersed in a container of PBF for 48 hours. The fixed lungs were then paraffin embedded and 4 μm sections were collected and stained with Hematoxylin and Eosin (H & E) for analysis. The mean linear intercept (*L*_m_), an indicator of mean alveolar diameter, was calculated essentially as previously described [33–35], counting the intercepts from 20 non-overlapping fields of lung parenchyma per animal at 200X magnification. The *L*_m_ was determined by counting the number of times that lines of the reticulum intercepted the alveolar walls and calculated using the equation: *L*_m_ = *L*_total_ / *NI*. *L*_total_ represented the sum of all segments of the reticulum, and *NI* is the average number of times the lines intersected the walls of the alveoli. The *L*_m_ values were expressed in microns (μm). The number of alveolar spaces per mm^2^ was also calculated from 20 non-overlapping areas of lung tissue from each mouse using Pannormic SCAN 150 (Perkin Elmer)(Results shown in the Supporting Information Section). The results of these two analyses are very similar, although it should be noted that the MLI is inversely correlated with the number of alveolar spaces / mm^2^.

### Murine model of influenza infection following cigarette smoke exposure

This procedure was undertaken with prior approval for this specific study obtained from the University of Melbourne’s Animal Ethics Committee (AEC, Melbourne, Australia) and experiments were carried out at the University of Melbourne by experienced and appropriately trained staff members. Throughout the study, mice were monitored daily and weighed at regular intervals. At the termination of the study, mice were euthanized by intraperitoneal injection of sodium pentobarbitone. Male BALB/c mice (sourced from Animal Resource Center, Canning Vale, Western Australia; male mice were used in this study to eliminate any effect regarding the estrous cycle, which may have an effect on viral exacerbations) received daily administrations of either vehicle, Compound C (10 mg/kg) or dexamethasone (1 mg/kg) by oral gavage (days 1–8). On days 2–5, mice were exposed to room air or cigarette smoke (3 x 45 minutes exposures per day). On day 6 of the study, mice were inoculated with 10^4.5^ pfu of influenza A via transnasal administration. Naïve mice or smoke control mice were not infected with flu, but instead received diluent. On day 9, bronchoalveolar lavage was performed and the lung tissue was isolated. Cytokines in the BAL fluid were determined using the BioPlex System (Bio-Rad Laboratories, Inc., Irvine, CA, USA) as per the manufacturer’s instructions. Quantitation of infective virus in the lungs of experimental mice was carried using an in vitro culture method. The right lung lobe was weighed, homogenized in 1 ml of DMEM media and centrifuged (2000 rpm, 5 minutes, 4°C). The supernatant was added to cultures of MDCK canine kidney cells (ATCC) seeded into 6-well plates, at 4 log dilutions. After an hour incubation at 37°C, the monolayers were overlayed with a warm solution of agar/media to form a plug that covered the monolayer. The cells were incubated at 37°C for two days and fixed for 24 hours in 2% paraformaldehyde. The agar plugs were carefully removed, and the cell monolayers were stained with crystal violet (0.5% w/v in methanol) and plaques were enumerated using a light box. Viral titer was expressed as PFU/g of tissue.

### Statistics

The data are presented as the mean values ± SEM, unless otherwise noted. The statistical difference between data sets was determined by one-way analysis of variance (ANOVA) and either the Tukey-Kramer pair-wise test or Bonferroni’s post-test was used to determine statistical differences between groups (as noted in the Figure Legend). Statistical analysis was performed using GraphPad Instat 3 software (GraphPad Software, San Diego, CA, USA). *P* values <0.05 were considered statistically significant.

## Results

### MAP3K19 is overexpressed in the lungs of COPD patients

In an effort to understand transcriptional changes that occur due to COPD, we initially compared lung RNA samples from healthy individuals to samples obtained from COPD patients and analyzed gene expression patterns by micro-array analysis. This study revealed that a novel kinase, MAP3K19, was upregulated in the lungs of COPD patients. We confirmed these initial findings by RT-qPCR analysis, which showed that MAP3K19 mRNA expression is 2.5–7 fold greater in the lungs of COPD patients (Fig 1A). To determine if any region of the lung had a greater expression of MAP3K19 in COPD patients, we assayed MAP3K19 mRNA expression in the trachea, large airways and small airways from 3 patients. In two of the three patients tested, the small airways showed the highest levels MAP3K19 expression, whereas the trachea and large airways displayed significantly lower amounts (Fig 1B). However, in the third patient (Patient 2 in Fig 1B), MAP3K19 expression was high in the trachea, significantly higher in the large airways and lowest in the small airways, demonstrating patient-to-patient variability. As macrophages are one of the main cell types that express MAP3K19 [28], we next determined whether the gene is overexpressed in pulmonary macrophages of COPD patients. Macrophages were purified from bronchoalveolar lavage of normal patients, healthy smokers and Gold stage I & II COPD patients, RNA was isolated and subjected to RT-qPCR analysis. This analysis revealed that five of the six COPD patients examined had significantly higher levels of MAP3K19 expression in BAL macrophages compared to non-diseased patients (Fig 1C), consistent with the RT-qPCR results of whole lung.

To investigate this further, normal human lung and biopsies from COPD patients were examined by IHC for MAP3K19 staining. As observed previously, MAP3K19 in normal human lung was found in ciliated bronchial epithelial cells, alveolar and interstitial macrophages, some type II pneumocytes and neutrophils (Fig 2A) [28]. In contrast, most of the cells composing the thickened epithelial layer of the large airways from COPD patients expressed MAP3K19, with the nuclear staining more intense than in the cytoplasm. Inflammatory cells, such as macrophages and lymphocytes in the lamina propria, were also strongly positive for MAP3K19 expression (Fig 2B, left and right panels). Finally, most of the cells composing the submucosal glands from COPD patients were positive for MAP3K19 expression (Fig 2B, center panel). Taken together, these results strongly suggest that COPD patients have elevated pulmonary levels of MAP3K19.

### MAP3K19 expression is induced by cell stress

**Fig 3:**
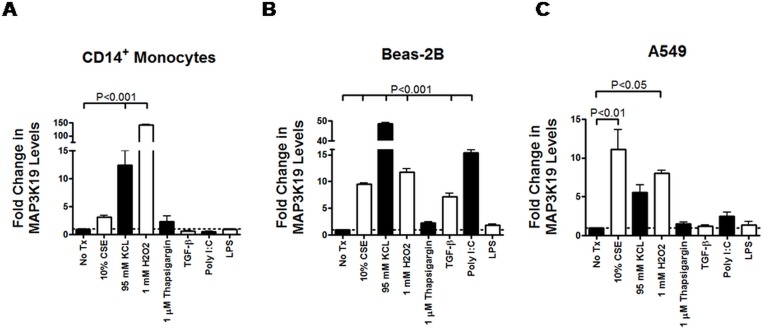
MAP3K19 is induced by the environmental stressors cigarette smoke extract, osmotic stress and oxidative stress. Freshly isolated CD14^+^ peripheral blood monocytes, A549 and Beas-2B pulmonary epithelial cell lines were incubated with 10% cigarette smoke extract, 95 mM KCl, 1 mM H_2_O_2_, 1 μM thapsigargin, 1 ng/ml TGF-β1, 1 ug/ml Poly I:C and 1 ug/ml LPS for 12 twelve hours. RNA was harvested, and the level of MAP3K19 mRNA expression was measured by RT-qPCR. All samples were normalized to GAPDH, and the results are expressed as fold change relative to the untreated (No Tx, denoted by the dotted line) sample. The RT-qPCR was run in duplicate, and the mean ± SEM are shown. This experiment was repeated two independent times and a representative experiment is shown. Similar results were obtained when either β-actin or 18s ribosomal RNA genes were used as reference genes. Cell viability, as determined by an XTT assay, was not affected by the different noxious stimuli over the course of the 12 hour experiment. THP-1 and U937 cells also yielded similar results to those shown for A549, Beas-2B and CD14^+^ peripheral blood monocytes. Statistical significance was determined by ANOVA analysis followed by Tukey-Kramer pair-wise tests and P<0.05 was considered significant.

MAP3K19 expression had previously been shown to be upregulated in rice plants subjected to salt stress [29]. As COPD is thought to result from repeated insults to the lung primarily from cigarette smoke, we investigated the ability of different cell stressors to induce MAP3K19 in cells. Examining either primary CD14^+^ peripheral blood monocytes or the pulmonary epithelial cell lines Beas-2B and A549, we found increased expression of MAP3K19 mRNA in cells cultured with 10% cigarette smoke extract (CSE), particularly in the epithelial cell lines (Fig 3B and 3C). Further, osmotic stress in the form of 95 mM KCl or oxidative stress (1 mM H_2_O_2_) strongly increased MAP3K19 transcription in all three cell types, while endoplasmic reticulum (ER) stress induced by thapsigargin was not as potent. Similar results were also observed for the THP-1 and U937 monocytic cell lines (dns). LPS had a negligible effect, whereas the TLR 3 ligand poly I:C caused a strong response in Beas-2B cells only. Interestingly, TGF-β1 treatment also increased MAP3K19 levels in Beas-2B cells. Treating the cells with other cytokines, such as IFN-ɣ, TNF-α or IL-4/IL-13 had a negligible effect on MAP3K19 gene expression levels (S1 Fig). These results suggested that MAP3K19 is induced when cells experienced environmental stress, and implicated this kinase in an evolutionarily conserved stress response pathway.

### Expression of MAP3K19 induced CXCL-8, CCL-7 and CCL-20 chemokine expression and activation of NF-kB

**Fig 4:**
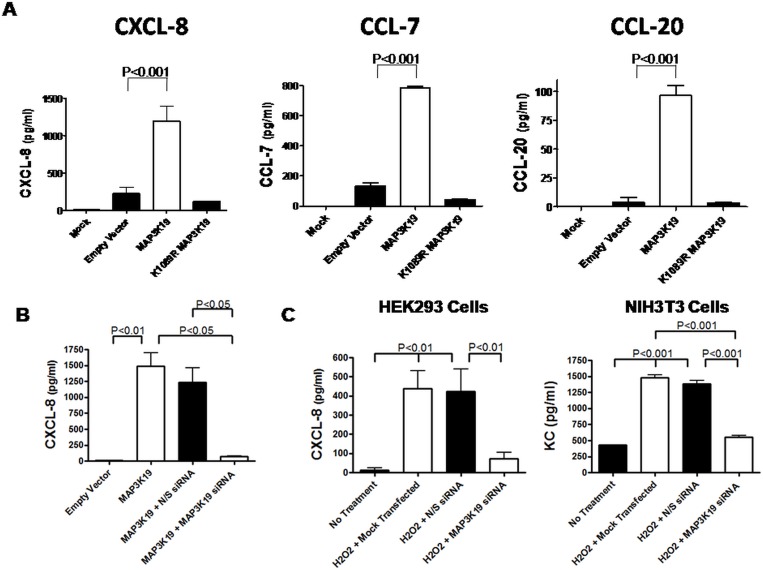
Transfection of HEK293 cells with MAP3K19 or treatment with H_2_O_2_ induces production of CXCL-8, CCL-20 and CCL-7 via a MAP3K19-dependent mechanism. (A) HEK293 cells were transiently transfected with 5 μg of either MAP3K19, a kinase inactive version of MAP3K19 containing a K1089R mutation, an empty vector or mock transfected. After 24 hours, the supernatants were assayed by ELISA for CXCXL-8, CCL-7 and CCL-20. The results shown are the mean of triplicates ± SEM. A representative experiment is shown, and the CXCL-8 was repeated over 20 independent times, CCL-7 and CCL-20 experiments were repeated a minimum of three times. Similar results were observed with HeLa, A549, THP-1, U937 and NIH3T3 cells. (B) HEK293 cells were transfected with empty vector or 5 μg of MAP3K19 in the absence or presence of 5 nM non-sense siRNA or MAP3K19 siRNA. The supernatants were assayed for CXCL-8 production by ELISA after 24 hours. A representative of three independent experiments is shown, and the results depict the mean of triplicate samples ± SEM. (C) HEK293 or NIH3T3 cells were transfected with 5 nM of human or mouse anti-MAP3K19 siRNA or non-sense siRNA, or mock transfected. After 18 hours, the cells were treated with 500 μM H_2_O_2_ for an additional 24 hours. At that point, the supernatants were assayed for CXCL-8 production (HEK293 cells) or KC production (NIH3T3 cells) by ELISA. The results of triplicate samples are shown as the mean ± SEM. The statistical significance was determined by ANOVA analysis followed by Tukey-Kramer pair-wise tests. Each arm of this experiment was repeated a minimum of two independent times, and a representative experiment is shown.

Although it was recently documented that MAP3K19 played a role in TGF-β-mediated signal transduction [28], we searched for a more relevant function with respect to COPD and cell stress. MAP3K19 was transfected into numerous cell types, such as THP-1 and HEK293, and ELISA analysis of cell supernatants revealed that the transfected cells produced significant amounts of CXCL-8 (IL-8), CCL-7 (MCP-3) and CCL-20 (MIP-3α) compared to empty vector transfected cells or cells transfected with a mutated kinase dead construct, K1089R MAP3K19 (Fig 4A). This was a surprising but very relevant finding, particularly as CXCL-8 is a powerful neutrophil chemoattractant linked to COPD pathology [25, 26] and CCL-20 has been implicated in attracting dendritic cells to the epithelium and adventitia of the small airways of COPD patients [36]. To further test whether MAP3K19 was responsible for the chemokine production, MAP3K19 was co-transfected with either non-sense siRNA or anti-MAP3K19 siRNA and as a read out CXCL-8 production was measured. As shown in Fig 4B, co-transfection of the specific MAP3K19 siRNA inhibited CXCL-8 production, whereas the non-sense siRNA had minimal effect. As H_2_O_2_ treatment induced MAP3K19 expression (Fig 3), we also noticed that H_2_O_2_ treatment elicited CXCL-8 production in both human HEK293 cells and murine NIH3T3 cells. We next asked whether the induction of MAP3K19 in the H_2_O_2_-treated cells could be involved in the pathway resulting in the production of either CXCL-8 in HEK293 cells or the mouse homologue of CXCL-8, KC in NIH3T3 cells, by transfecting the cells first with either non-sense siRNA or anti-MAP3K19 siRNA. In both cell types examined, transfection of the specific siRNA significantly reduced H_2_O_2_-induced chemokine levels, whereas the non-sense siRNA had no effect (Fig 4C). As only one copy of MAP3K19 exists per genome (G. Manning, pers. commun.), these findings demonstrated a critical role for this kinase in coordinating stress stimuli with a pro-inflammatory response.

**Fig 5:**
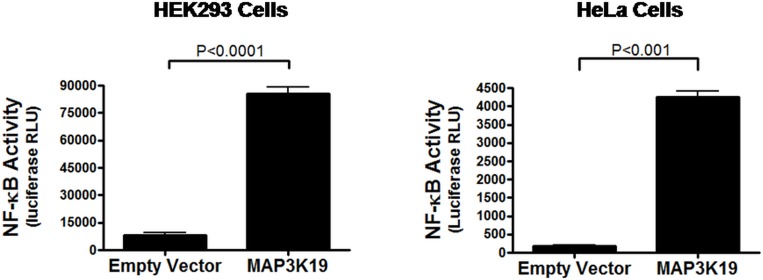
MAP3K19 induces NF-κB-mediated transcription. HEK293 or HeLa cells were transiently transfected with either a MAP3K19 expression plasmid or an empty vector plasmid and a plasmid containing an NF-κB binding element linked to a luciferase reporter gene. After 24 hours, the cells were assayed for luciferase levels, indicative of NF-κB transcriptional activation. The amount of luciferase activity as measured by relative light units (RLU) is shown ± SEM, and representative experiments from a minimum of three independent experiments are shown.

**Fig 6:**
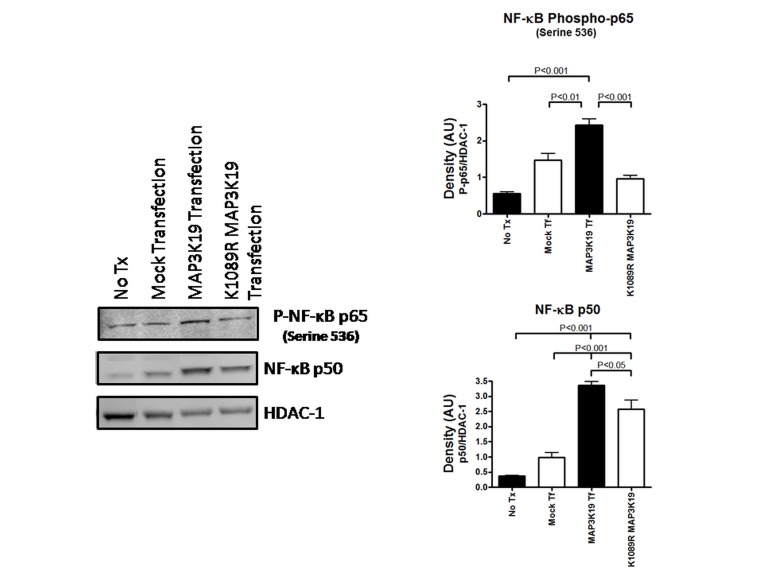
MAP3K19 promotes the nuclear translocation of NF-κB (phospho-p65/p50) in transfected HEK293 cells. HEK293 were transfected with a MAP3K19 expression plasmid, a plasmid encoding a kinase inactive version of MAP3K19 due to a K1089R mutation, or an empty vector plasmid (mock transfection), and after 24 hours nuclear extracts were isolated. Nuclear lysates were examined for phospho-NF-κB p65 (serine 536) and NF-κB p50 subunit levels, and the blots were re-probed with HDAC-1 to show equal loading. Densitometry was performed comparing phospho-p65/HDAC-1 levels or p50/HDAC-1 levels. This experiment was repeated three independent times and a representative experiment is shown.

To understand the molecular basis of how MAP3K19 induces pro-inflammatory chemokines we examined the transcription factor NF-κB, because the promoter for CXCL-8 contains an NF-κB element that is required for activation in all cell types examined [37]. HEK293 or HeLa cells were co-transfected with either a MAP3K19 expression plasmid or an empty vector together with a reporter construct containing an NF-κB binding element linked to a luciferase gene. After 24 hours, the luciferase activity was determined, and this analysis showed that MAP3K19 transfection resulted in NF-κB driven transcription and this corresponded to CXCL-8 production (Fig 5). This was further examined by Western analysis of nuclear lysates of MAP3K19 or K1089R MAP3K19 transfected HEK293 cells. Transfection of only the kinase active MAP3K19 plasmid was able to promote the nuclear translocation the phospho-(Ser536)-p65 NF-κB subunit and the p50 NF-κB subunit, but the mock transfected or the K1089R kinase dead MAP3K19 transfected cells exhibited only marginally increased levels of either NF-κB subunit (Fig 6). Consistent with the NF-κB reporter experiments, serine 536 phosphorylation of p65 correlates with NF-κB transcriptional activation [38]. In total, these data illustrated that MAP3K19 promoted NF-κB activation, which resulted in transcriptional activation and the production of the pro-inflammatory chemokines CXCL-8, CCL-20 and CCL-7.

### siRNA-mediated inhibition of MAP3K19 blocked cigarette smoke-induced pulmonary inflammation

**Fig 7:**
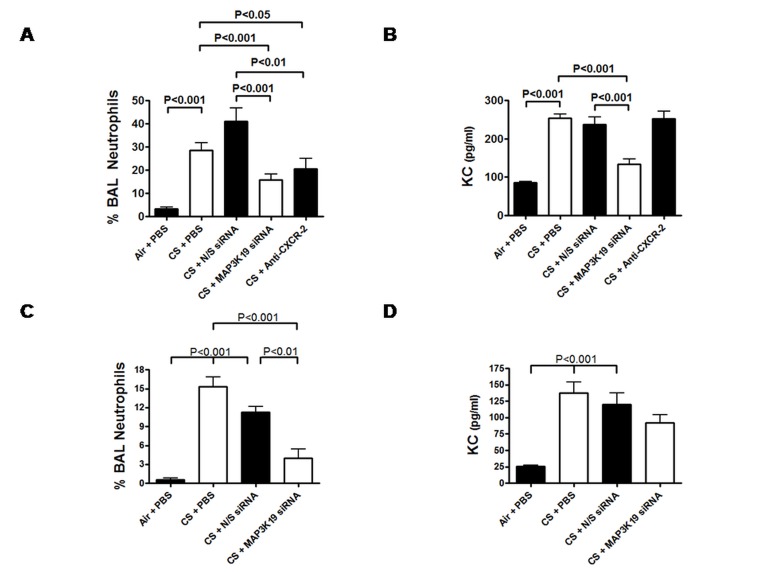
MAP3K19 siRNA inhibits cigarette smoke-induced pulmonary inflammation. The acute model of cigarette smoke exposure subjected mice to cigarette smoke (7 cigarettes over a 1.75 hour period) on days 1 & 2, and dosed with either PBS, non-sense siRNA (100 μg in 50 μl volume), MAP3K19 siRNA or the CXCR-2 antagonist SB 265610 (10 mg/kg, p.o.) on days 1, 2 & 3 (n = 5 mice per treatment group). The inflammatory response was quantitated by performing bronchoalveolar lavage on day 4, counting the total number cells in the BAL, and enumerating the different cell types by morphological features. (A) MAP3K19 siRNA reduced the neutrophil influx compared to vehicle or non-sense siRNA treated mice. (B) ELISA analysis of the BAL fluid showed a significant decrease in KC, the murine homologue of CXCL-8, in MAP3K19 siRNA treated animals. This experiment was repeated three times and the average of 2 representative experiments is shown. (C & D) Mice treated with MAP3K19 siRNA had decreased pulmonary inflammation in a subchronic cigarette smoke exposure model. Mice (5 per cohort) were exposed to cigarette smoke on days 1, 2, 5–9, and 12–14, and dosed with PBS, non-sense siRNA or MAP3K19 siRNA on days 6–14. The mice were examined for pulmonary inflammation of day 15, and the percent of neutrophils in the BAL fluid was calculated (C), and the BAL fluid KC levels were determined by ELISA. This experiment was repeated two independent times with similar results, and a representative experiment is shown. In both experiments, the numbers shown are the mean ± SEM. Statistical differences between groups was determined by ANOVA analysis followed by the Tukey-Kramer pair-wise tests, and P<0.05 was considered significant. In both the acute and subchronic experiments, a cohort of mice were exposed to air instead of cigarette smoke, and treated with PBS delivered i.t., as a control.

Because MAP3K19 is (1) overexpressed in COPD lungs, (2) expressed in pulmonary macrophages and bronchial epithelial cells, (3) induced by CSE and other environmental stressors, and (4) drives the neutrophil chemoattractant CXCL-8 expression, we wanted to examine the role of MAP3K19 in murine models of COPD. This was tested in an acute cigarette smoke exposure model where mice were treated with intra-tracheally (i.t.) delivered siRNA. To demonstrate that the siRNA was able to reach the lower airways, anti-MAP3K19 siRNA was labeled with Cy3 dye and administered to mice intra-tracheally twice over a 24 hour period. As observed by histological analysis, the i.t. administered siRNA was dispersed throughout the lung lobe (S2 Fig) [39]. Next, mice were treated either intra-tracheally with phospho-buffered saline (PBS, siRNA vehicle), non-sense siRNA or MAP3K19 siRNA, or an orally delivered CXCR-2 antagonist compound SB265610, as a positive control, on days 1, 2 and 3, and subjected to cigarette smoke on days 1 and 2. Western analysis of the lung tissue showed that MAP3K19 siRNA effectively reduced protein levels of the kinase (S3 Fig). Analysis of the bronchoalveolar lavage fluid showed that the smoke treatment increased the number of neutrophils approximately 15-fold and treatment with non-sense siRNA had a negligible effect (Fig 7A). However, anti-MAP3K19 siRNA significantly reduced the pulmonary neutrophilia to an even greater extent than the anti-CXCR-2 control compound. ELISA analysis of the BAL fluid for KC levels showed that only the anti-MAP3K19 siRNA significantly reduced the chemoattractant levels, as the CXCR-2 inhibitor had no effect, as expected (Fig 7B). This reduction of KC chemokine levels is consistent with the decrease in neutrophils observed.

To expand these observations even further, mice were subjected to a subchronic cigarette smoke exposure protocol, in which they were exposed to cigarette smoke for 10 sessions over a fourteen day period. They were treated with either PBS, non-sense siRNA or MAP3K19 siRNA delivered intra-tracheally from day 6–14, in a therapeutic treatment dosing regimen. As shown in Fig 7C, treatment with MAP3K19 siRNA decreased the amount of pulmonary neutrophils in the BAL fluid by approximately 65%, the roughly the same amount as observed in the acute model. Additionally, we noticed a concomitant decrease in the KC levels in the BAL fluid that mirrored the effect on pulmonary neutrophils (Fig 7D). Collectively, these siRNA studies were the first evidence in an animal model that inhibition of MAP3K19 could provide therapeutic benefit to cigarette smoke induced inflammation.

### Inhibition of MAP3K19 attenuated pulmonary inflammation and airway tissue destruction in murine models of COPD

**Fig 8:**
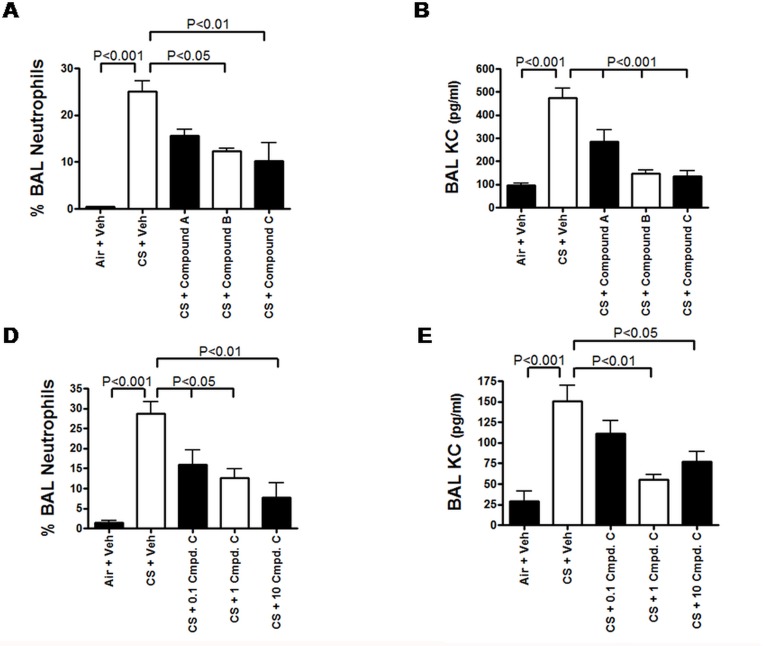
Small molecule inhibitors of MAP3K19 attenuate cigarette smoke-mediated pulmonary inflammation in an acute model of COPD. Mice were dosed with vehicle or Compound A, B or C (10 mg/kg, p.o.) daily on days 1–3, and exposed to cigarette smoke on days 1 & 2 (n = 5 mice per treatment group). Inflammation was measured on day 4. (A) Inhibition of MAP3K19 reduced BAL neutrophils. (B) MAP3K19 inhibitors reduced KC levels in the BAL fluid. (C) Dose response analysis of Compound C. Mice were treated in the acute model of cigarette smoke-induced pulmonary inflammation with either 10, 1 or 0.1 mg/kg Compound C delivered orally on days 1–3. Results show a dose related decrease in pulmonary neutrophils and KC levels. This experiment was repeated over 20 times with multiple small molecule inhibitors of MAP3K19 with similar results, and at least 3 times with the compounds shown here. Results from a representative experiment are shown. The results shown are the mean of each cohort ± SEM, and significant differences between groups was determined by ANOVA analysis followed by the Tukey-Kramer pair-wise tests.

We have identified several novel, small molecule inhibitors of MAP3K19 kinase activity that are orally active, selective and have a range of potencies. We first tested three of these inhibitors, Compounds A, B and C (dosed at 10 mg/kg, p.o.) in an acute cigarette smoke model of COPD. Differential cell counts of the bronchoalveolar lavage fluid from animals treated with air revealed that 1.54% of the cells recovered in the BAL fluid were neutrophils, with macrophages being the predominant cell type recovered and a small number of lymphocytes present. In contrast, cigarette smoke exposed animals had 25% neutrophils in the BAL. When smoke-exposed animals were treated, Compound A treated mice showed a 37.6% decrease in BAL neutrophils (Compound A, 15.6% BAL neutrophils), Compound B had a 51% decrease (12.3% neutrophils in BAL) and Compound C demonstrated a 60% decrease in BAL neutrophils (10.2% neutrophils in BAL) (Fig 8A). Analysis of KC levels in the BAL fluid showed a significant reduction in all cohorts treated with compounds compared to vehicle treated animals, particularly with the Compound B and C treated groups (Fig 8B), and this correlated with the neutrophil influx into the lung. (A table showing all the cell numbers examined in the BAL fluid is displayed in S5 Table). Histological examination of lung sections showed markedly reduced inflammation in the compound treated lung sections when compared to the smoke and vehicle treated mice (dns). Dose response analysis of Compound C (administered 10, 1, and 0.1 mg/kg) showed a dose-dependent decrease in BAL neutrophils and KC levels (Fig 8C and 8D) in the acute cigarette smoke model. Finally, we investigated whether there was a difference in the pulmonary inflammation caused by the research cigarettes versus a commercially available brand. These results demonstrated that the amount of neutrophils in the BAL was very similar (research cigarette –21.8%, 3.5 x 10^4^ neutrophils; commercial cigarette– 23.2%, 3.0 x 10^4^ neutrophils) and the ability for Compound C to impact the pulmonary neutrophilia was also comparable (research cigarette + Compound C– 11.2%, 1.2 x 10^4^ neutrophils; commercial cigarette + Compound C– 11.7%, 1.5 x 10^4^ cells) (S4 Fig).

**Fig 9:**
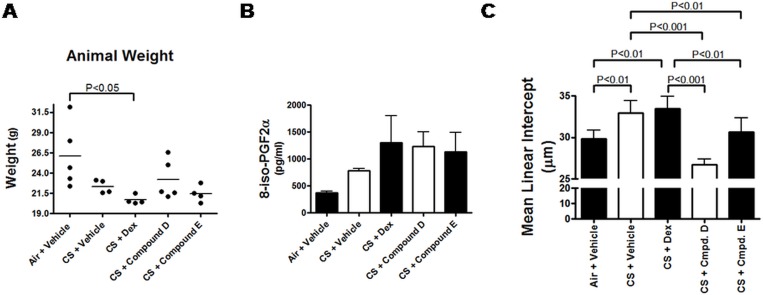
Airway tissue destruction induced by chronic cigarette smoke exposure is blocked by MAP3K19 inhibitors administered in a therapeutic dosing regimen. Mice were exposed to cigarette smoke or air 5 days per week from days 1–156, and dosed with either vehicle, Compound D or E (10 mg/kg, p.o.), or dexamethasone (1 mg/kg, i.p.) daily from days 78–156 in a therapeutic dosing model (5 mice per group). At the termination of the study, the mice were weighed, serum was obtained and the lungs were isolated and gently fixed in phosphate buffered formalin to carefully preserve the lung structure, prior to histology. Panel (A) shows the final weight of each animal, (B) shows the level of prostaglandin F2α in the serum samples, and (C) shows the mean linear intercept analysis, which is an indicator of mean alveolar diameter. The mean linear intercept values, or *L*_m_ values, were expressed in microns (μm) (mean of 20 non-overlapping sections of lung parenchyma per animal were measured, results are expressed as the mean ± SEM, as detailed in the Materials & Methods). The greater the *L*_m_ value is an indication of greater alveolar space and correlates with airspace destruction, and is consistent with emphysematous changes. This can be observed on the histological analysis in Fig 10.

**Fig 10:**
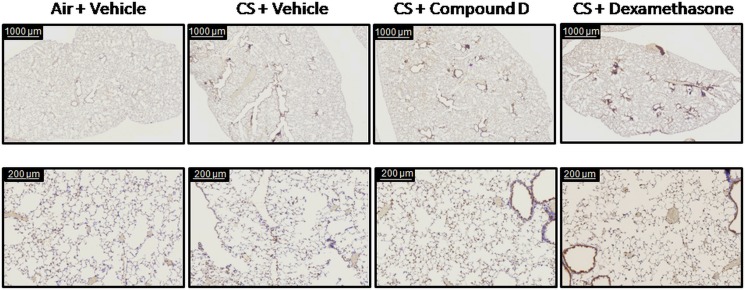
Histological analysis shows that cigarette smoke-induced airspace enlargement is abrogated by MAP3K19 inhibitors. Lung sections from mice that underwent the chronic cigarette smoke exposure model were stained with a rabbit polyclonal anti-MAP3K19 antibody, RabK19, which is shown in brown. No statistical difference was observed in the MAP3K19 staining between the different cohorts. In the animals exposed to cigarette smoke, lymphoid follicles are clearly visible, and most of the cells composing the follicles express MAP3K19. The sections were counterstained with hematoxylin.

Having demonstrated that inhibition of MAP3K19 can impact the inflammation observed in a four day acute cigarette smoke model, we next wanted to assess whether we could impact the parenchymal changes in the lung following a five month chronic model of COPD with therapeutic dosing of the animals. Animals received cigarette smoke for 5 days per week for eleven weeks, and starting on day 78, the mice were randomized into 4 cohorts and treated daily with (1) vehicle, (2) Compound D, (3) Compound E, or (4) dexamethasone. A fifth cohort received air and vehicle. On day 156, the mice were sacrificed and the lungs harvested *en bloc*. The animals exposed to cigarette smoke all had decreased body weights compared to the air exposed mice, however the weight loss was less marked in the MAP3K19 inhibitor treated mice, particularly those treated with the more potent Compound D (Fig 9A). Additionally, all the smoke treated mice had elevated serum levels of 8-oxo prostaglandin F2α, which is a marker of oxidative stress-induced lipid peroxidation caused by chronic cigarette smoke exposure (Fig 9B). Lung histological sections, stained with CD45/B220 showed the presence of lymphoid follicles in all cigarette smoke exposed cohorts, but not in air treated animals (S5 and S6 Figs). Lymphoid follicles, consisting of predominantly B lymphocytes, have been characterized in the lungs of patients with severe COPD [40, 41], and independently demonstrated that the chronic cigarette smoke exposure had a profound effect on the mice. Further examination of histological sections revealed airspace enlargement, as measured by mean linear intercept analysis, was greatest in the CS + vehicle and dexamethasone treated mice compared to the MAP3K19 inhibitor treated or air treated mice (Figs 9C and 10, S7 Fig). These results were consistent with findings in human COPD patients in that glucocorticoid therapy does not block the development of emphysema [13]. The lung structural alterations, consistent with the development of emphysematous changes, were significantly ameliorated in mice receiving Compound D, where the mean linear intercept value, an indicator of mean alveolar diameter, was similar to the air + vehicle treated mice (Fig 9C). Similar results were observed measuring the number of alveolar spaces per mm^2^ (S7 Fig). Together with the increased body weight, these results show that inhibition of MAP3K19 by a small molecule inhibitor via therapeutic dosing regimen can provide clear clinical benefit over a twenty-two week period of chronic cigarette smoke exposure.

### Blockade of MAP3K19 activity reduced inflammation and viral load in an acute viral exacerbation model of COPD

**Fig 11:**
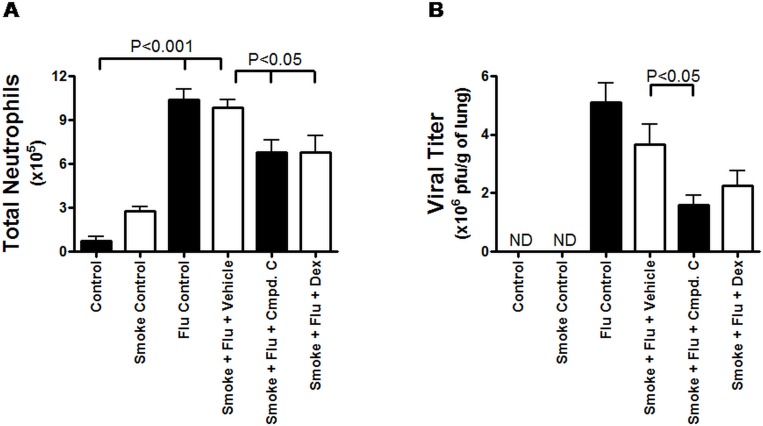
MAP3K19 inhibition blocks pulmonary neutrophilia and reduces viral load in an acute viral exacerbation model of COPD. Mice were orally dosed with either vehicle, dexamethasone (1 mg/kg), Compound C (10 mg/kg) on days 1–8 of the study. The mice were exposed to cigarette smoke (3 x 45 minute exposures per day) on days 2–5, and inoculated with 10^4.5^ pfu of influenza A virus via transnasal administration on day 6. On day 9, bronchoalveolar lavage was performed and the total number of neutrophils in the BAL fluid was enumerated (A). The lung tissue was isolated and the amount of infective virus in the lungs was quantitated (B). The viral titer was expressed as PFU/g of lung tissue. The number of mice in each cohort was as follows: (1) control mice, treated with sham + diluent + vehicle (n = 5); (2) smoke control mice received smoke + diluent + vehicle (n = 5); (3) flu control mice were treated with sham + flu + vehicle (n = 5); (4) smoke + flu + vehicle (n = 8); (5) smoke + flu + Compound C (n = 8); and (6) smoke + flu + dexamethasone (n = 8). ND–not detected. Statistically significant differences between groups was determined by ANOVA analysis followed by Boniferroni’s post-test as detailed in the Materials and Methods.

**Fig 12:**
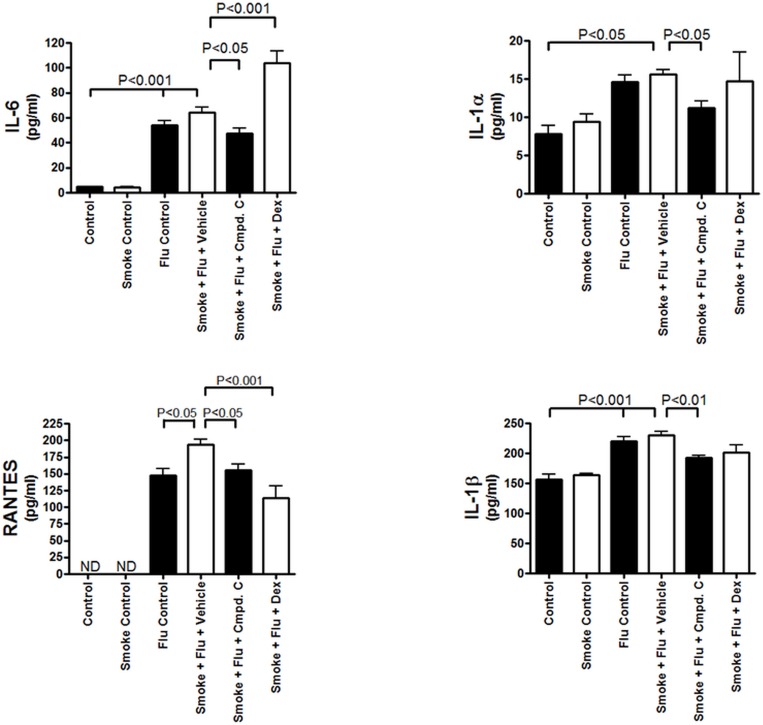
Inhibition of MAP3K19 decreases pro-inflammatory cytokines in the BAL fluid of mice subjected to an acute viral exacerbation model of COPD. Mice were treated in the viral exacerbation model and on day 9 underwent bronchoalveolar lavage. The lavage fluid was assayed for the cytokines IL-1α, IL-1β, IL-6 and RANTES. Each sample from each mouse was examined in triplicate. The mean ± SEM is shown. ND–not detected. Differences between groups were determined to be significant by ANOVA analysis followed by Boniferroni’s post-test.

Respiratory viral infections are a common and dangerous co-morbidity of COPD [14, 15]. We assessed the effect of MAP3K19 inhibition on various parameters in a murine model of influenza infection following cigarette smoke exposure. Similar to the results observed with the acute cigarette smoke model, mice that received only cigarette smoke had an increase in BAL neutrophils. However, the amount of neutrophils increased almost 3.5 fold upon infection with influenza virus (Fig 11A). Significantly, mice treated with either Compound C or dexamethasone had an approximate 32% decrease in the amount of BAL neutrophils. An examination of the viral titer showed that Compound C caused a 56.8% decrease in the viral titer, which was more effective than dexamethasone treatment (38.8% decrease) (Fig 11B). This was particularly surprising when considered in light of the decreased pulmonary neutrophils in Compound C treated animals. Furthermore, there was not a significant difference in the number of BAL macrophages in the smoke + flu animals treated with vehicle, Compound C or dexamethasone (dns). An examination of cytokines in the BAL fluid revealed that Compound C treatment caused significant decreases in the amount of IL-1α, IL-1β, IL-6 and RANTES, and in all cases except RANTES, Compound C treatment resulted in a greater decrease than dexamethasone (Fig 12). Collectively, these results demonstrate that MAP3K19 inhibition can effectively block many of the inflammatory processes involved in the pathogenesis of COPD, including those associated with a viral exacerbation.

## Discussion

Our results of a limited patient population demonstrated by either RNA expression analysis or histological examination that MAP3K19 RNA levels or protein levels appeared to be overexpressed in COPD patients versus healthy patients. Although we are in the process of expanding these findings in a larger patient population, these results are quite striking and consistent with an important role of MAP3K19 in the pathogenesis of COPD.

MAP3K19 is an evolutionarily conserved kinase with homologues found in all multi-cellular animals and some protist species (G. Manning, pers. commun.). Our results here, coupled with the observation of Schmid et al. [29] that show MAP3K19 is upregulated in rice plants subjected to salt stress, suggest that this kinase is part of an evolutionarily conserved pathway involved in the cellular response to environmental stress. The predominant cell types expressing MAP3K19 in human and mouse lung and trachea include bronchial epithelial cells and macrophages, and these cell types are among the first sites of contact for environmental stimuli with airway tissue. Both of these cell types express numerous innate immune receptors geared toward recognizing and responding to microorganisms [42, 43]. However, the respiratory tissue is also subjected to environmental stressors, such as oxidative stress from both exogenous sources (eg. cigarette smoke, air pollution, ozone) and endogenous sources (eg. activated neutrophils, macrophages, eosinophils and epithelial cells) [44]. This type of stress not only leads to MAP3K19 upregulation (Fig 3), but also to MAP3K19-dependent NF-κB activation and pro-inflammatory chemokine production (Figs 4, 5 and 6). These observations suggest that MAP3K19 may be a key molecule in a conserved pathway governing pulmonary inflammation in response to various noxious stimuli in the lung.

In multiple murine models, we have demonstrated that the exposure of mice to cigarette smoke leads to the main neutrophil chemoattractant being produced in the lung, KC, and a concomitant increase in neutrophil recruitment [25, 26]. Using both siRNA and specific, orally bioavailable small molecule inhibitors of MAP3K19, we demonstrated that inhibition of MAP3K19 kinase activity resulted in decreased pulmonary neutrophilia and KC production (Figs 7 and 8). These decreases in pro-inflammatory mediators and cell types are thought to have a direct effect on obstructing the pathological process leading to airway tissue destruction that occurs in the lung parenchyma upon prolonged exposure to cigarette smoke (Figs 9 and 10). Additionally, increased TGF-β1 has been found in COPD patients, and some patients have been shown to have dysregulated TGF-β signaling [45, 46]. Furthermore, it has been reported that angiotensin receptor blockade abrogates cigarette smoke-induced lung injury by antagonism of TGF-β signaling [47]. It has already been shown that MAP3K19 can regulate TGF-β signaling and induced gene transcription by governing the nuclear translocation of the activated phospho-Smads [28]. This represents another possible avenue by which inhibition of MAP3K19 inhibition is acting to attenuate cigarette smoke-induced lung injury.

Another interesting observation is that mice treated with the MAP3K19 inhibitor also had significantly decreased viral load in the cigarette smoke viral exacerbation model (Fig 11B). This was surprising as the MAP3K19 inhibitor reduced the number of neutrophils in the lung and there was no effect on the amount of macrophages compared to the other smoke and flu treated cohorts (Fig 11A). Our preliminary results have demonstrated that inhibition of MAP3K19 had no effect on the respiratory burst or phagocytosis of macrophages (data not shown). However, recent results may help explain this finding, in which it was shown that decreased TGF-β levels following respiratory viral infection allow IFN-β levels to rise quickly, which leads to a decrease in virus production [48, C. Lloyd, pers. commun.]. It is plausible that MAP3K19 inhibition, which results in decreased TGF-β signaling, released the TGF-β-mediated constraint on IFN-β production. This could allow for more immediate and robust IFN-β production and a subsequent decrease in viral load. This hypothesis is currently being tested.

In conclusion, our data provide evidence that MAP3K19 acts as a molecular sensor of environmental stress, orchestrating a response coordinating the NF-κB pro-inflammatory pathway leading to chemokine secretion and neutrophil recruitment and the TGF-β pathway that impacts tissue remodeling and airspace enlargement. The restricted expression pattern and pathways the kinase plays a critical role in regulating, strongly suggest that inhibition of MAP3K19 may provide a multipronged therapeutic approach for the treatment of COPD.

## Supporting Information

S1 Fig The cytokines IFN-γ, TNF-α and IL-4 / IL-13 do not cause an upregulation of MAP3K19 expression in A549 cells. A549 cells were cultured with either IFN-γ, TNF-α or IL4 / IL-13 for 12 hours, and the RNA was assayed by RT-qPCR for expression of MAP3K19 and normalized to GAPDH. Untreated (No Tx) cells were arbitrarily assigned a value of one, and the MAP3K19 levels in all other samples were compared to that. Similar results were observed with Beas-2B and THP-1 cells.
(TIF)

S2 Fig siRNA delivered intra-tracheally was able to reach the lower airways of the lung. BALB/c mice received two intra-tracheal doses of either PBS or Cy3-labeled MAP3K19 siRNA (100 μg siRNA in 50 μl volume) 24 hours apart. One hour after the second dose, the mice were sacrificed, and the lungs were sectioned, counterstained with DAPI and visualized. The Cy3-labeled siRNA is visualized in gold staining.
(TIF)

S3 Fig MAP3K19 siRNA can knock down the pulmonary protein levels following intra-tracheal administration. Nuclear protein from lungs of siRNA treated mice in one of the experiments shown in Fig 7A & 7B was examined for MAP3K19 expression by Western analysis. The top panel shows the Western results for MAP3K19 expression and HDAC-1 as a loading control. The lower panel shows the results of densitometry. There is statistically significant increase in MAP3K19 expression in animals receiving cigarette smoke in the acute smoking model, most likely due to the influx of MAP3K19 expressing neutrophils and macrophages into the lung. Treatment with MAP3K19 siRNA results in a significant decrease in MAP3K19 expression compared to non-sense siRNA treated animals.
(TIF)

S4 Fig Research cigarettes and commercially available cigarettes show a similar amount of pulmonary neutrophilia, and inhibition by Compound C. Mice were treated in the acute cigarette smoke model using either research cigarettes (University of Kentucky) or a commercially available brand and treated with Compound C (10 mg/kg, p.o.).
(TIF)

S5 Fig Chronic cigarette smoke exposure caused the formation of lymphoid follicles. Lung sections from air treated and the different cigarette smoke treated cohorts were stained with the B lymphocyte marker CD45/B220. This analysis showed that all cohorts exposed to cigarette smoke over the five month period, but not air, developed lymphoid follicles in the lung parenchyma, a hallmark also observed in severe COPD patients. S5 Fig shows the lung histology from two mice in each of the following cohorts: (1) Air exposed + Vehicle treated, (2) CS (cigarette smoke) exposed + Vehicle treated and (3) CS (cigarette smoke) exposed + Dexamethasone treated.
(TIF)

S6 Fig Chronic cigarette smoke exposure caused the formation of lymphoid follicles. Lung sections from air treated and the different cigarette smoke treated cohorts were stained with the B lymphocyte marker CD45/B220. This analysis showed that all cohorts exposed to cigarette smoke over the five month period, but not air, developed lymphoid follicles in the lung parenchyma, a hallmark also observed in severe COPD patients. S6 Fig shows the lung histology from two mice in each of the following cohorts: (1) CS (cigarette smoke) exposed + Compound D treated and (2) CS (cigarette smoke) exposed + Compound E treated.
(TIF)

S7 Fig MAP3K19 inhibitors attenuate emphysematous changes in a chronic model of cigarette smoke exposure as measured by the number of alveolar spaces per mm^2^. This digitized analysis was previously shown to be directly comparable to the mean linear intercept analysis (Fig 9C), and quantitated the amount of airspace enlargement due to destruction of the lung parenchyma by cigarette smoke exposure. The greater number of alveolar spaces / mm^2^ is inversely correlated with airspace enlargement while the lower number of alveolar airspaces / mm^2^ correlated with airspace destruction, consistent with emphysematous changes. This can be observed on the histological analysis in Fig 10.
(TIF)

S1 Table Patient demographics from patients in Fig 1A. (TIF)

S2 Table Patient demographics from patients in Fig 1B. (TIF)

S3 Table Patient demographics from patients in Fig 1C. (TIF)

S4 Table Patient demographics from patients in Fig 2. (TIF)

S5 Table Cell counts showing a decrease in BAL neutrophil numbers and KC levels in Compound A, B and C treated mice. The raw cell numbers extrapolated for the graphs shown in Fig 8A and 8B.
(TIF)
