# Supplementary figures and images for: MAP3K19 Is Overexpressed in COPD and Is a Central Mediator of Cigarette Smoke-Induced Pulmonary Inflammation and Lower Airway Destruction
Source: PLoS One. 2016 Dec 9;11(12):e0167169. doi: 10.1371/journal.pone.0167169 (PMC5147866; doi:10.1371/journal.pone.0167169)

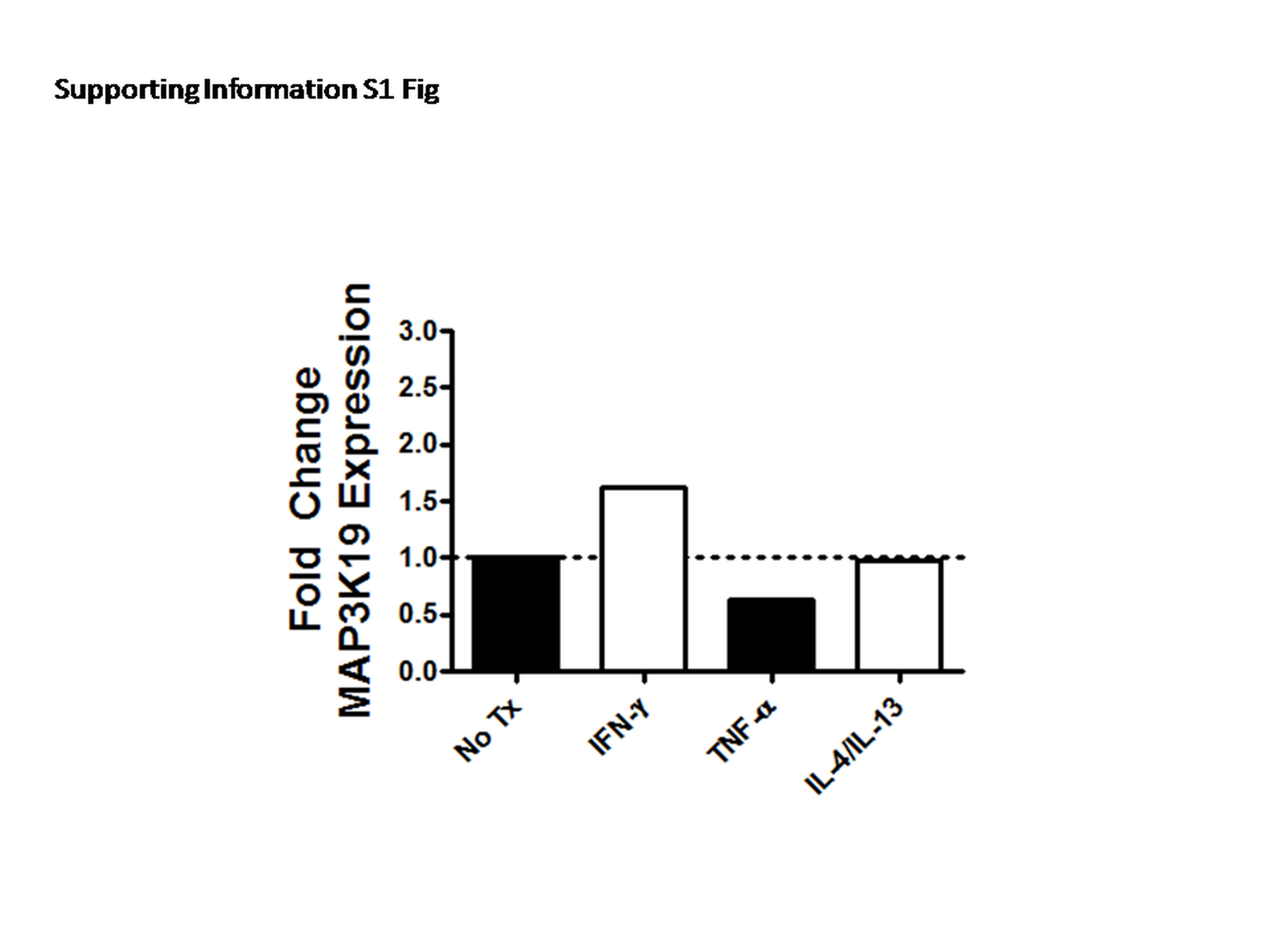

Supplement: S1 Fig — A549 cells were cultured with either IFN-γ, TNF-α or IL4 / IL-13 for 12 hours, and the RNA was assayed by RT-qPCR for expression of MAP3K19 and normalized to GAPDH. Untreated (No Tx) cells were arbitrarily assigned a value of one, and the MAP3K19 levels in all other samples were compared to that. Similar results were observed with Beas-2B and THP-1 cells. (TIF) [file pone.0167169.s001.tif]

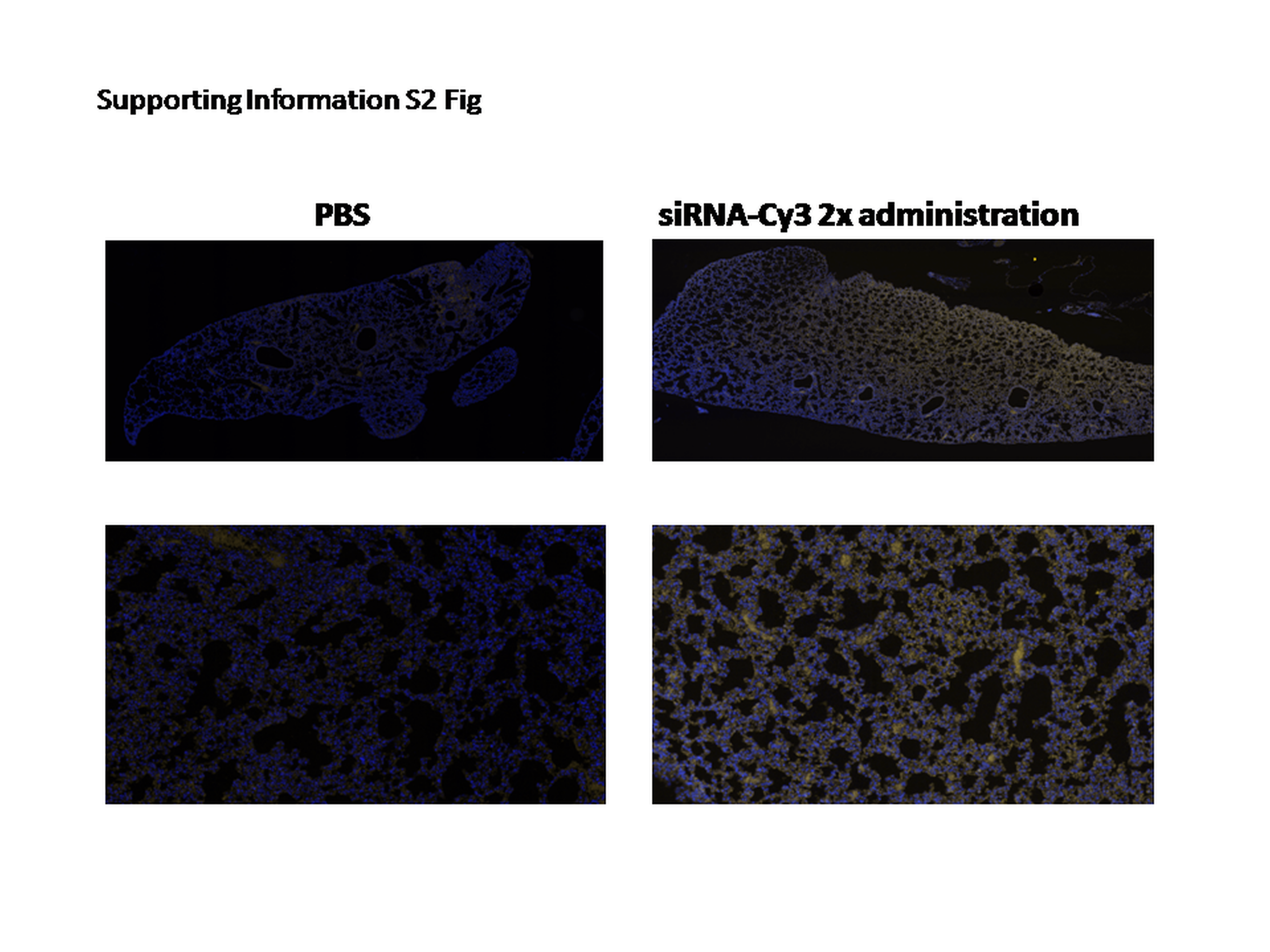

Supplement: S2 Fig — BALB/c mice received two intra-tracheal doses of either PBS or Cy3-labeled MAP3K19 siRNA (100 μg siRNA in 50 μl volume) 24 hours apart. One hour after the second dose, the mice were sacrificed, and the lungs were sectioned, counterstained with DAPI and visualized. The Cy3-labeled siRNA is visualized in gold staining. (TIF) [file pone.0167169.s002.tif]

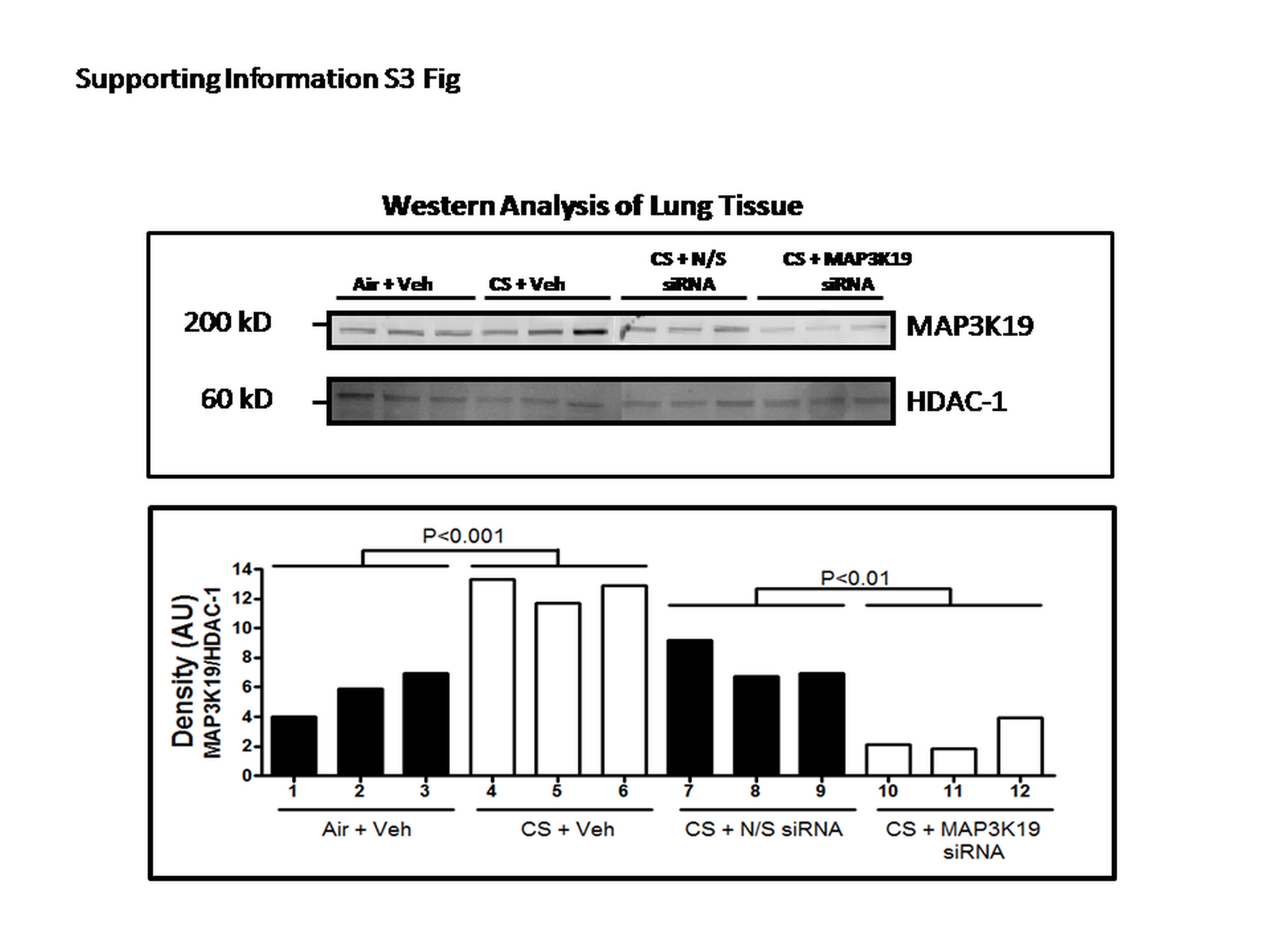

Supplement: S3 Fig — Nuclear protein from lungs of siRNA treated mice in one of the experiments shown in Fig 7A & 7B was examined for MAP3K19 expression by Western analysis. The top panel shows the Western results for MAP3K19 expression and HDAC-1 as a loading control. The lower panel shows the results of densitometry. There is statistically significant increase in MAP3K19 expression in animals receiving cigarette smoke in the acute smoking model, most likely due to the influx of MAP3K19 expressing neutrophils and macrophages into the lung. Treatment with MAP3K19 siRNA results in a significant decrease in MAP3K19 expression compared to non-sense siRNA treated animals. (TIF) [file pone.0167169.s003.tif]

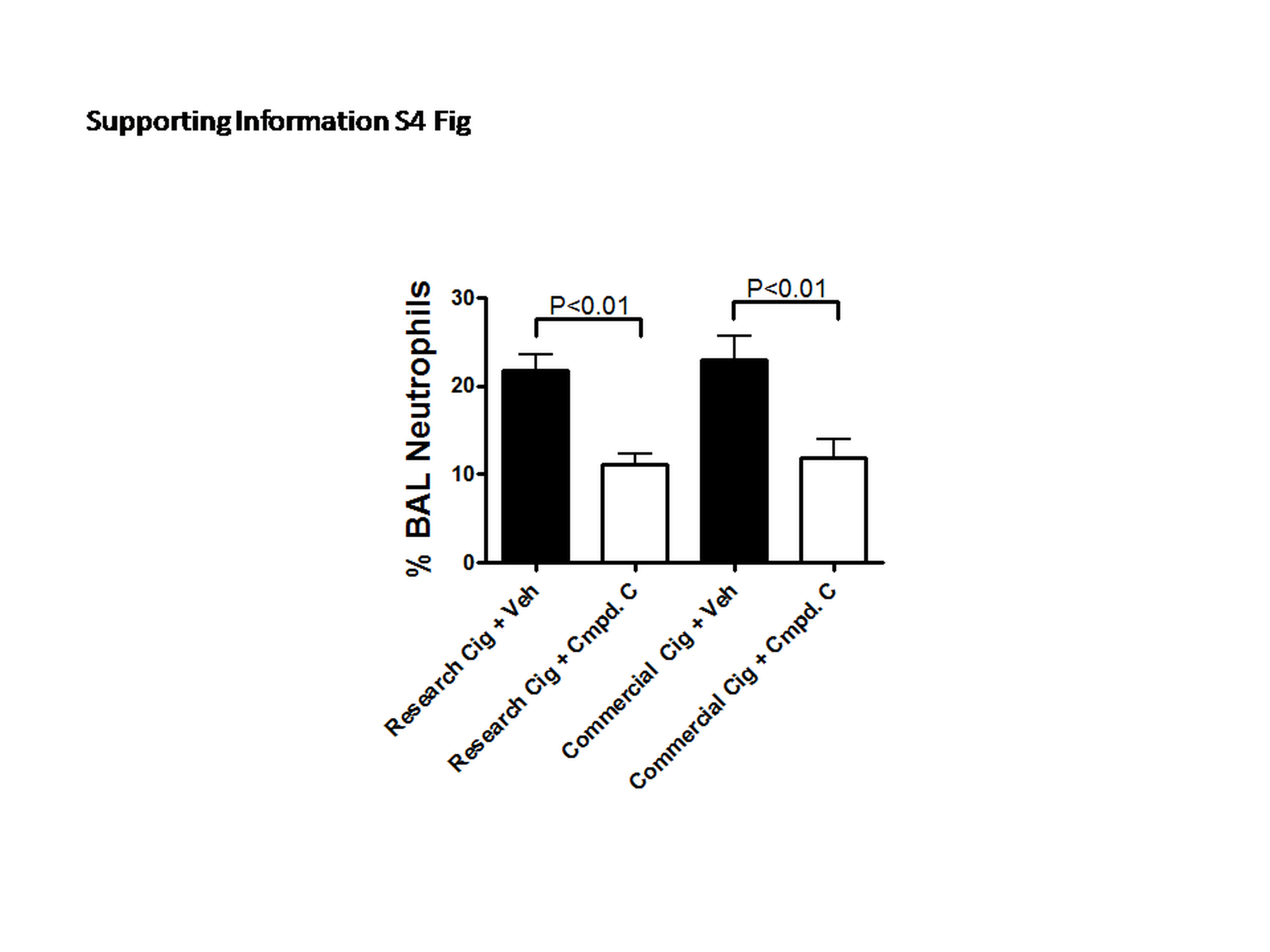

Supplement: S4 Fig — Mice were treated in the acute cigarette smoke model using either research cigarettes (University of Kentucky) or a commercially available brand and treated with Compound C (10 mg/kg, p.o.). (TIF) [file pone.0167169.s004.tif]

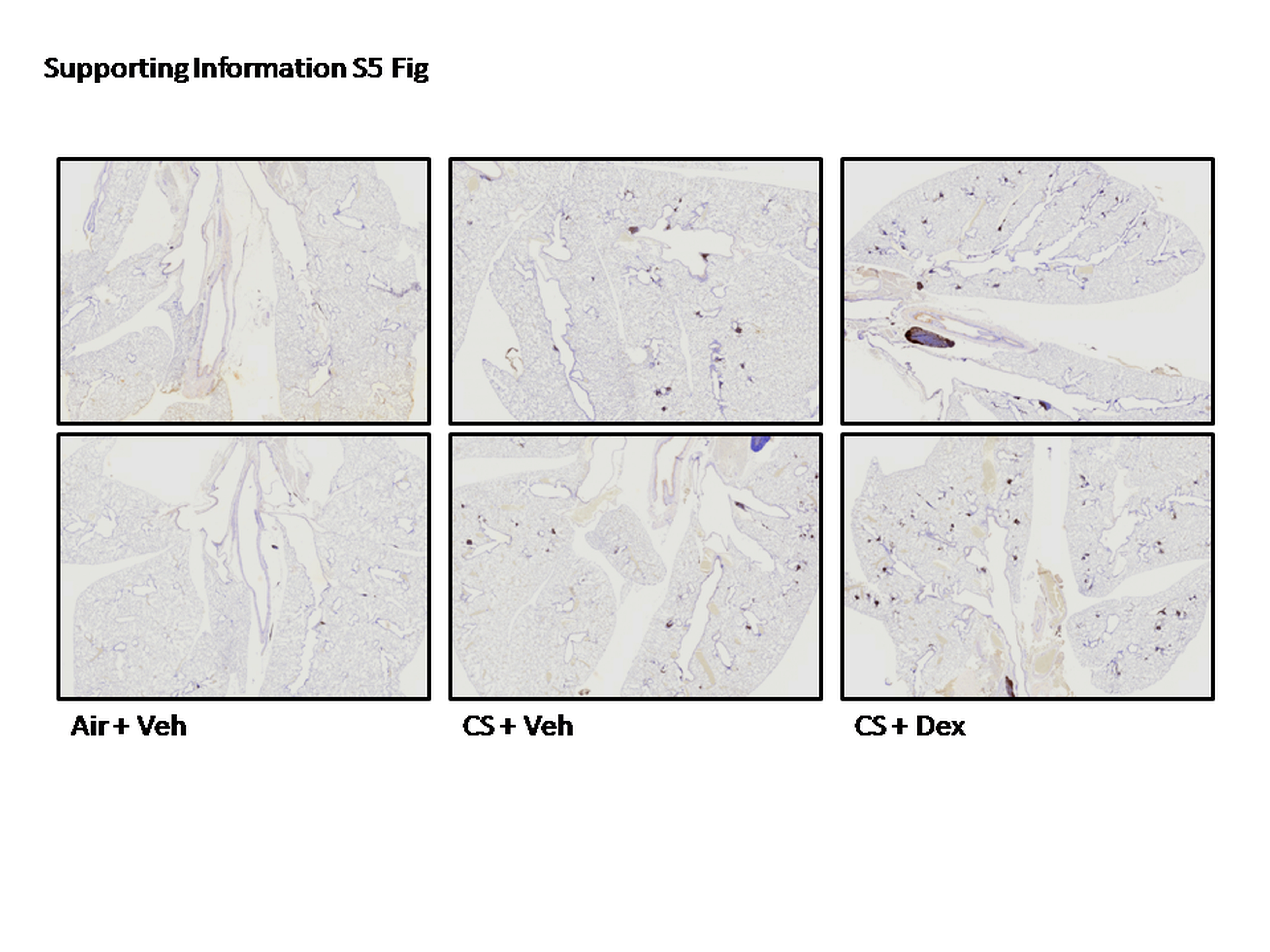

Supplement: S5 Fig — Lung sections from air treated and the different cigarette smoke treated cohorts were stained with the B lymphocyte marker CD45/B220. This analysis showed that all cohorts exposed to cigarette smoke over the five month period, but not air, developed lymphoid follicles in the lung parenchyma, a hallmark also observed in severe COPD patients. S5 Fig shows the lung histology from two mice in each of the following cohorts: (1) Air exposed + Vehicle treated, (2) CS (cigarette smoke) exposed + Vehicle treated and (3) CS (cigarette smoke) exposed + Dexamethasone treated. (TIF) [file pone.0167169.s005.tif]

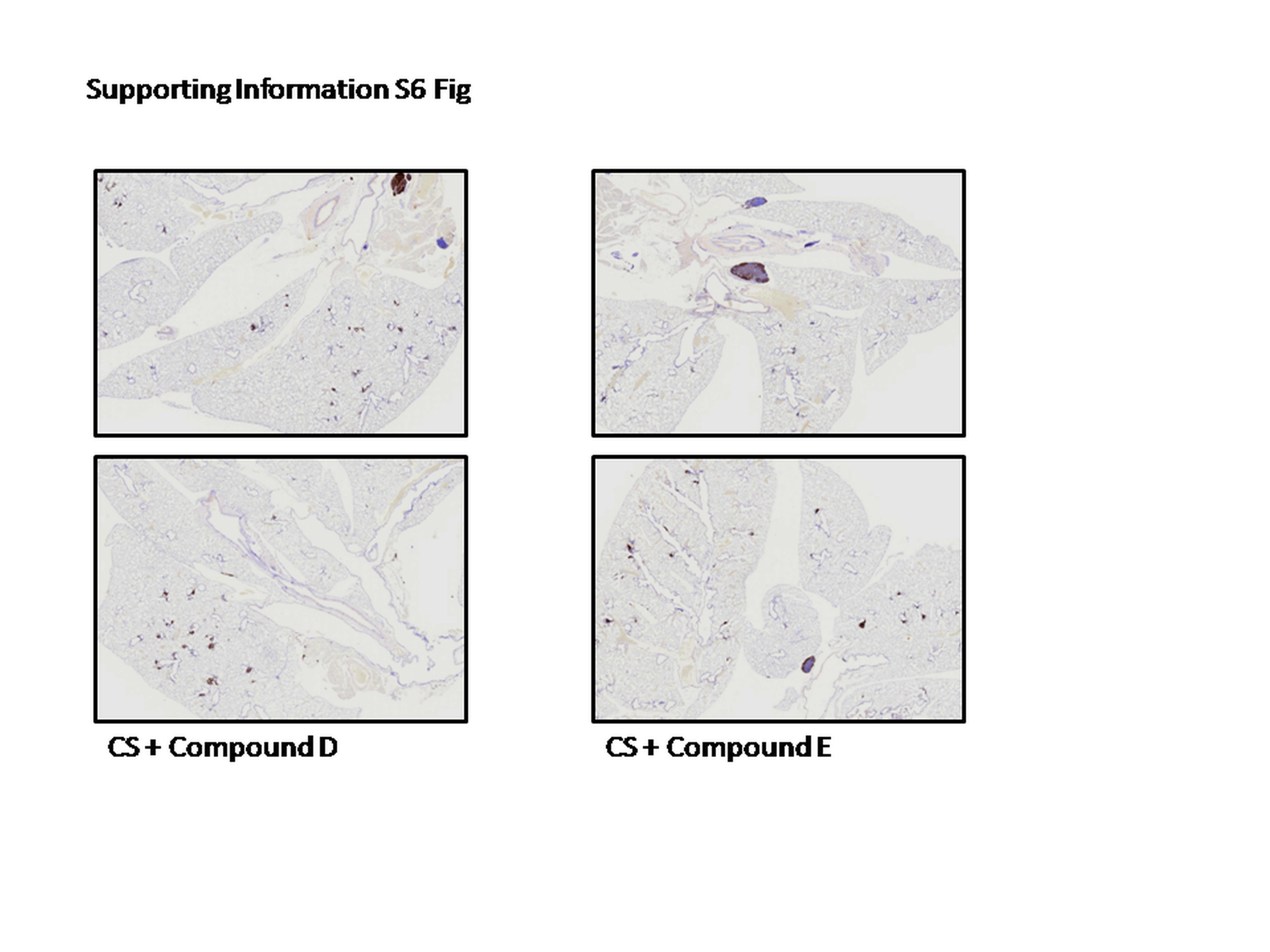

Supplement: S6 Fig — Lung sections from air treated and the different cigarette smoke treated cohorts were stained with the B lymphocyte marker CD45/B220. This analysis showed that all cohorts exposed to cigarette smoke over the five month period, but not air, developed lymphoid follicles in the lung parenchyma, a hallmark also observed in severe COPD patients. S6 Fig shows the lung histology from two mice in each of the following cohorts: (1) CS (cigarette smoke) exposed + Compound D treated and (2) CS (cigarette smoke) exposed + Compound E treated. (TIF) [file pone.0167169.s006.tif]

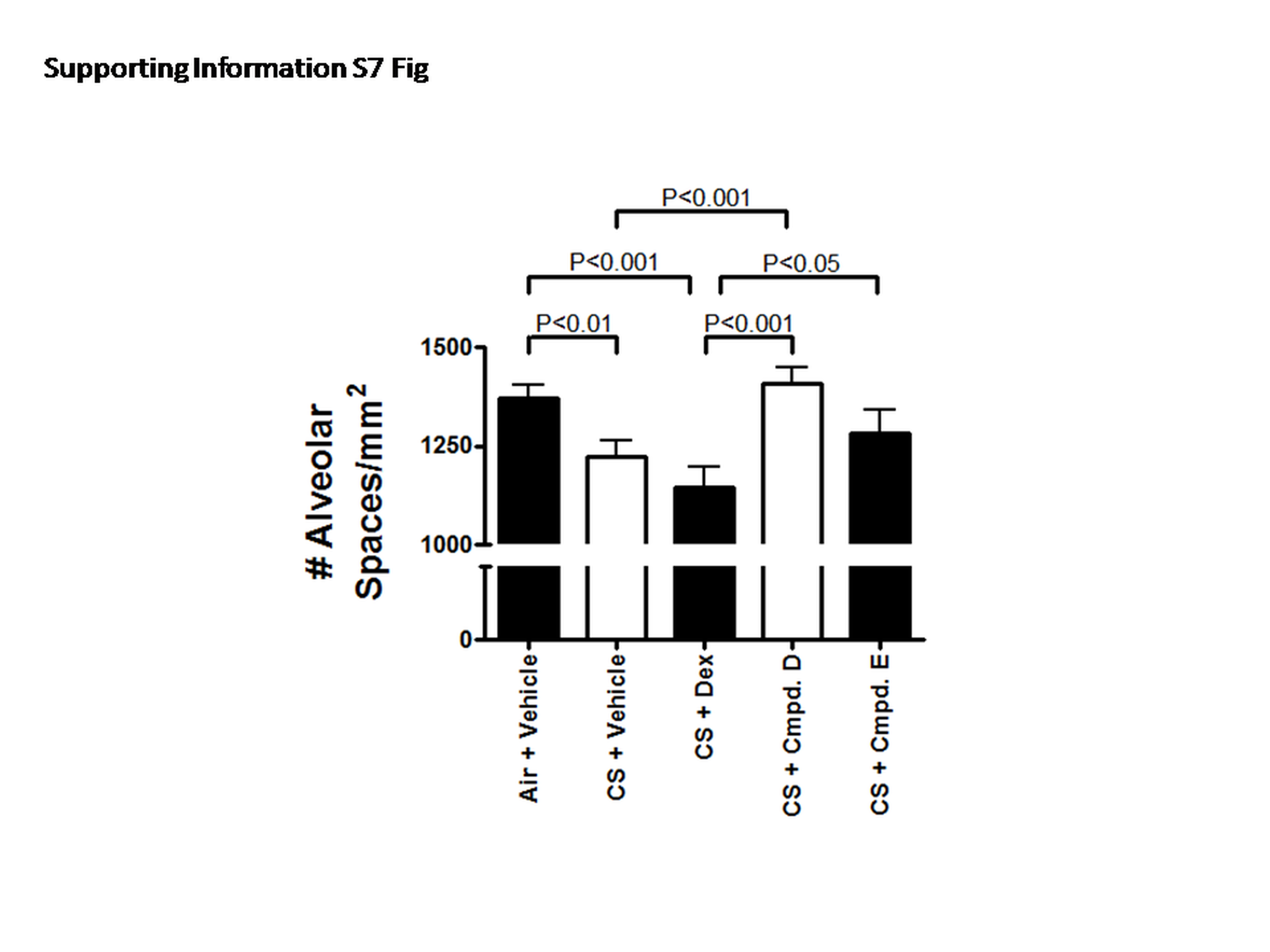

Supplement: S7 Fig — This digitized analysis was previously shown to be directly comparable to the mean linear intercept analysis (Fig 9C), and quantitated the amount of airspace enlargement due to destruction of the lung parenchyma by cigarette smoke exposure. The greater number of alveolar spaces / mm2 is inversely correlated with airspace enlargement while the lower number of alveolar airspaces / mm2 correlated with airspace destruction, consistent with emphysematous changes. This can be observed on the histological analysis in Fig 10. (TIF) [file pone.0167169.s007.tif]

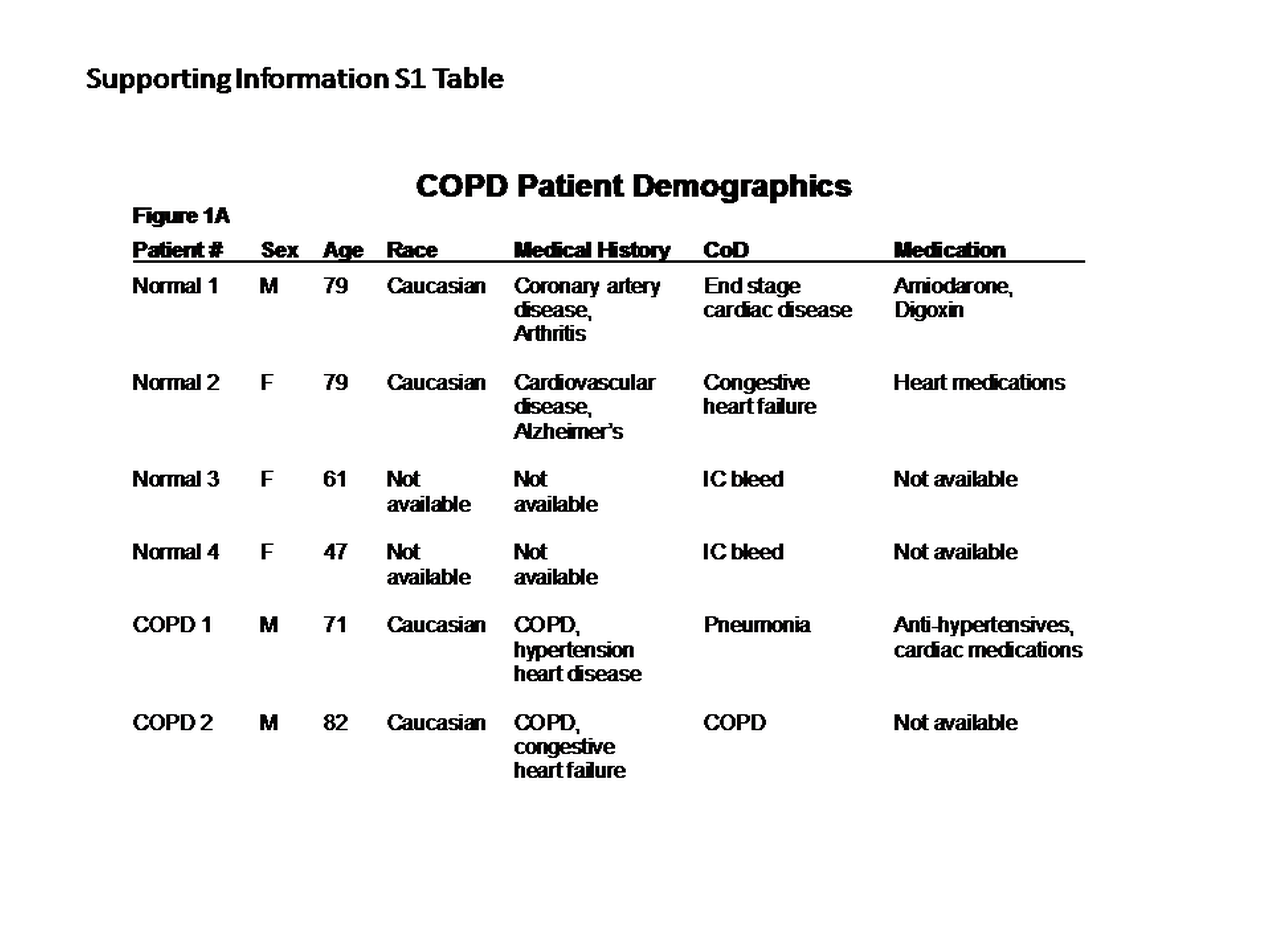

Supplement: S1 Table — (TIF) [file pone.0167169.s008.tif]

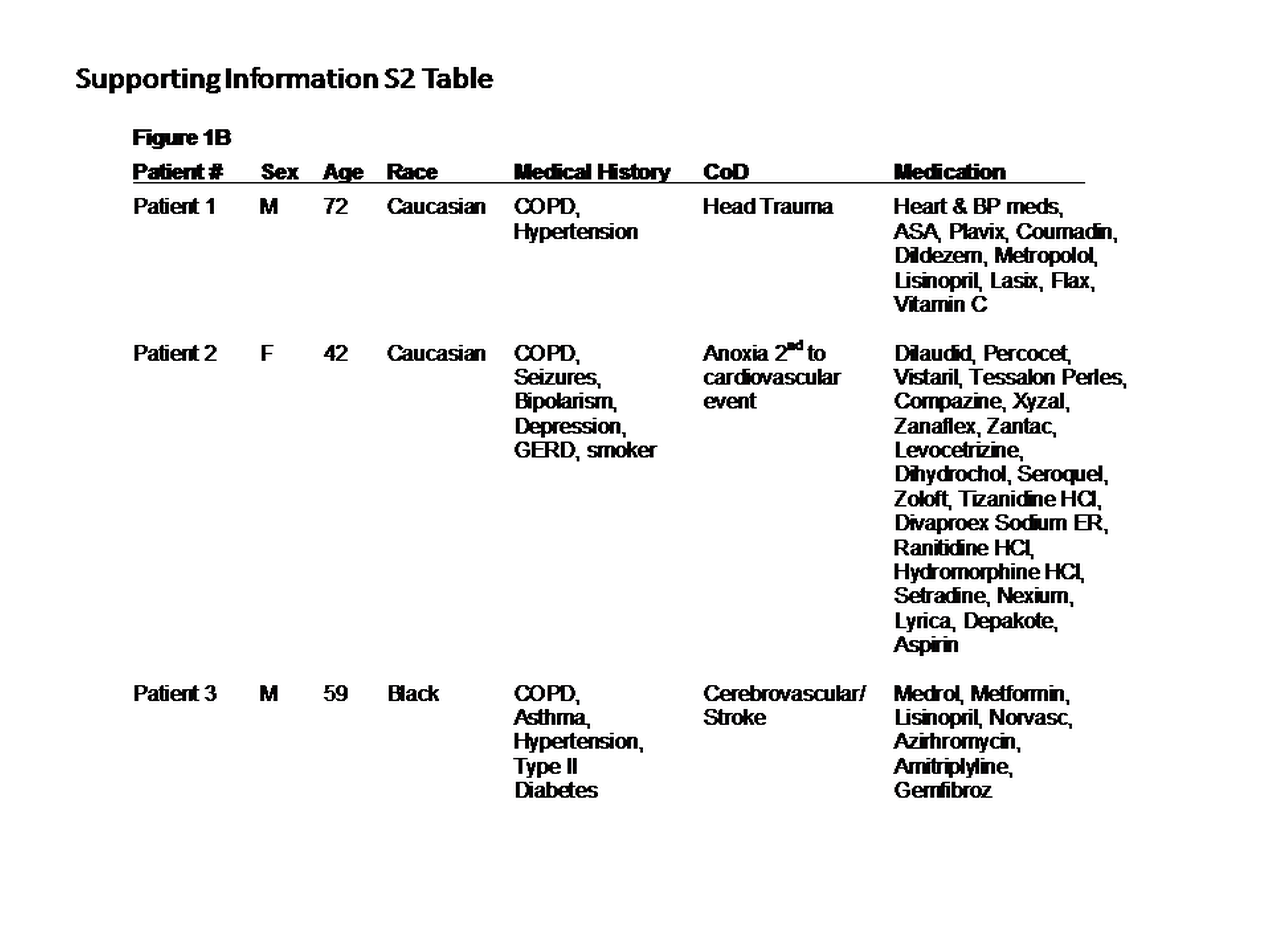

Supplement: S2 Table — (TIF) [file pone.0167169.s009.tif]

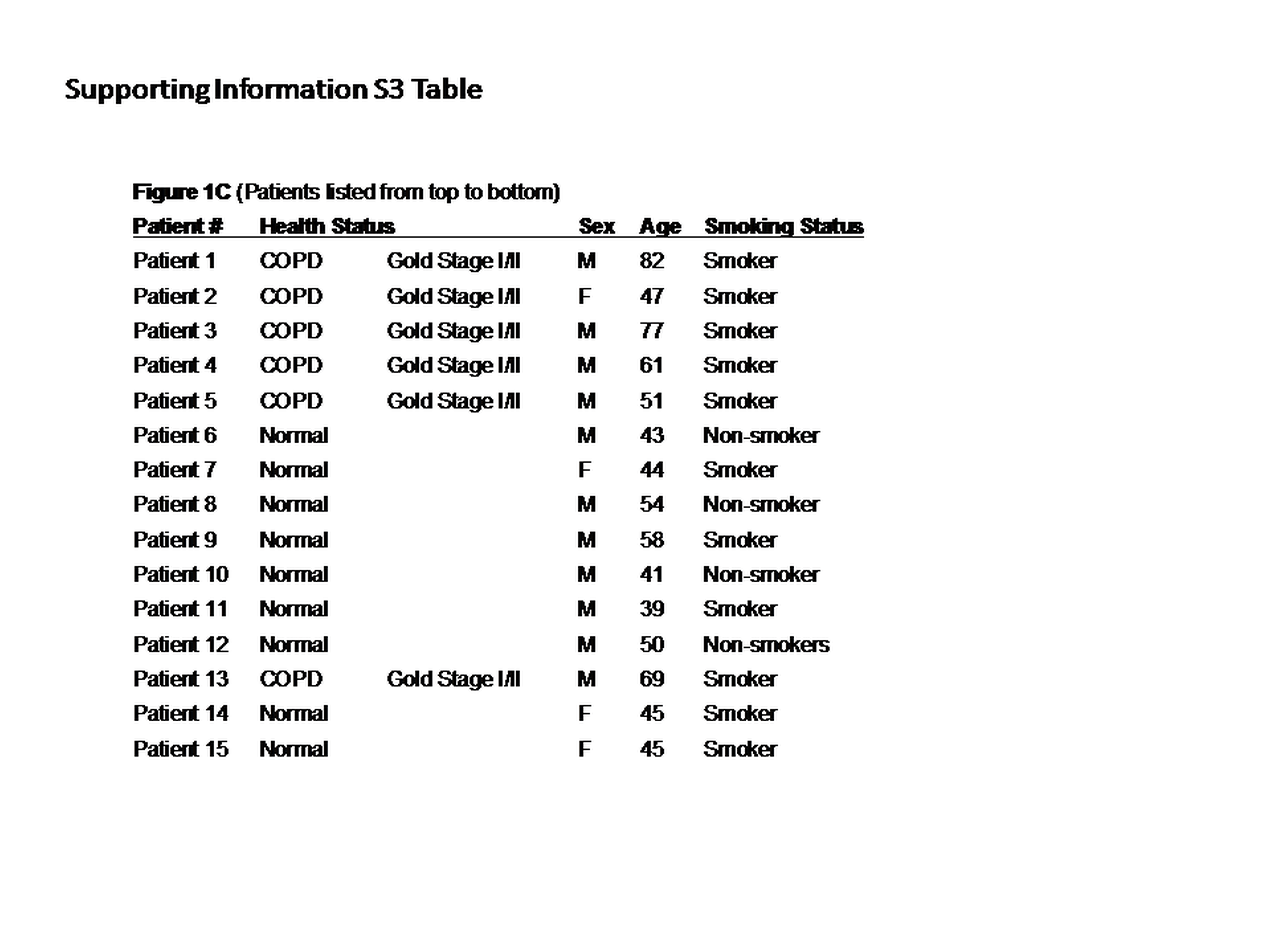

Supplement: S3 Table — (TIF) [file pone.0167169.s010.tif]

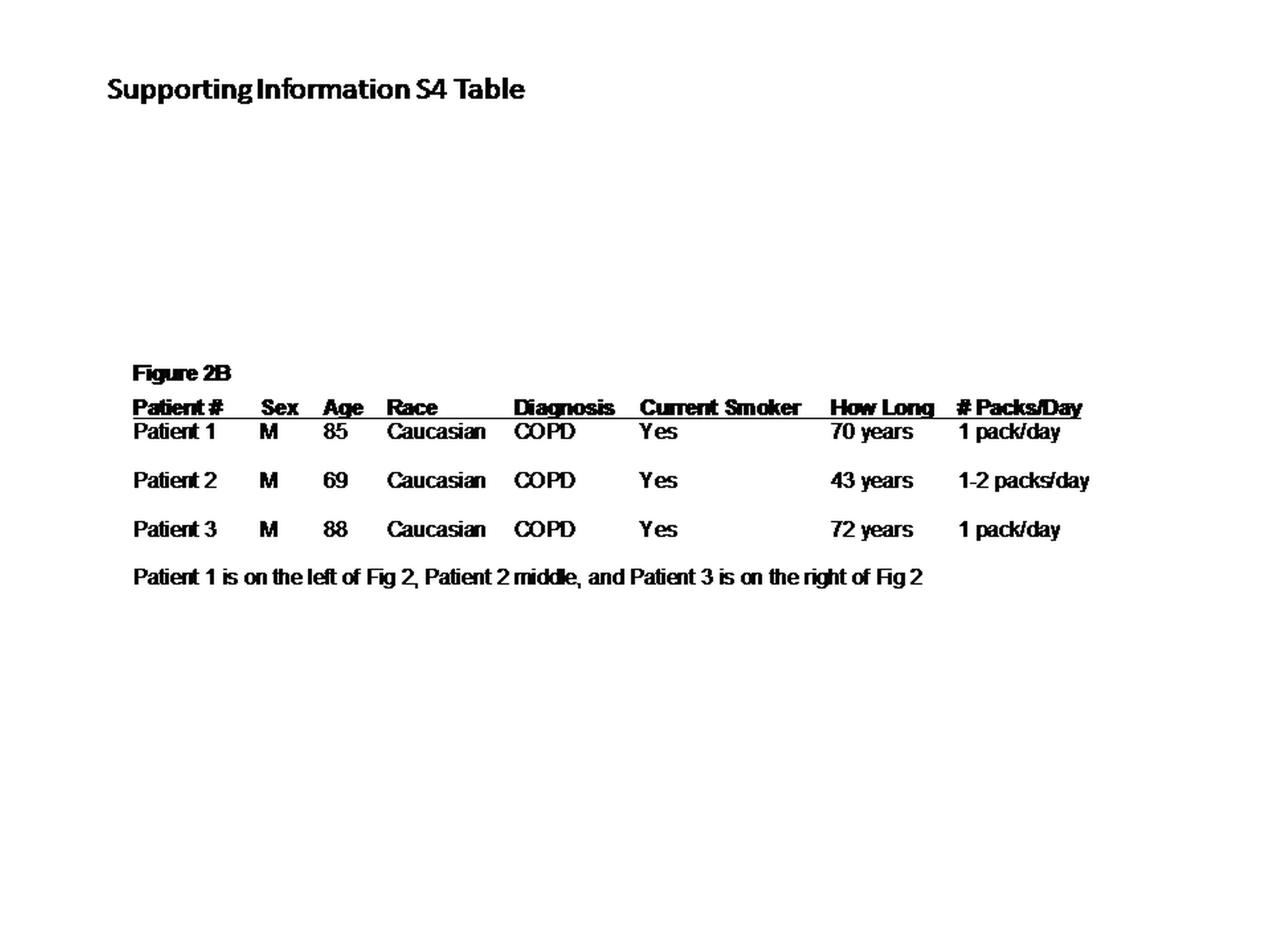

Supplement: S4 Table — (TIF) [file pone.0167169.s011.tif]

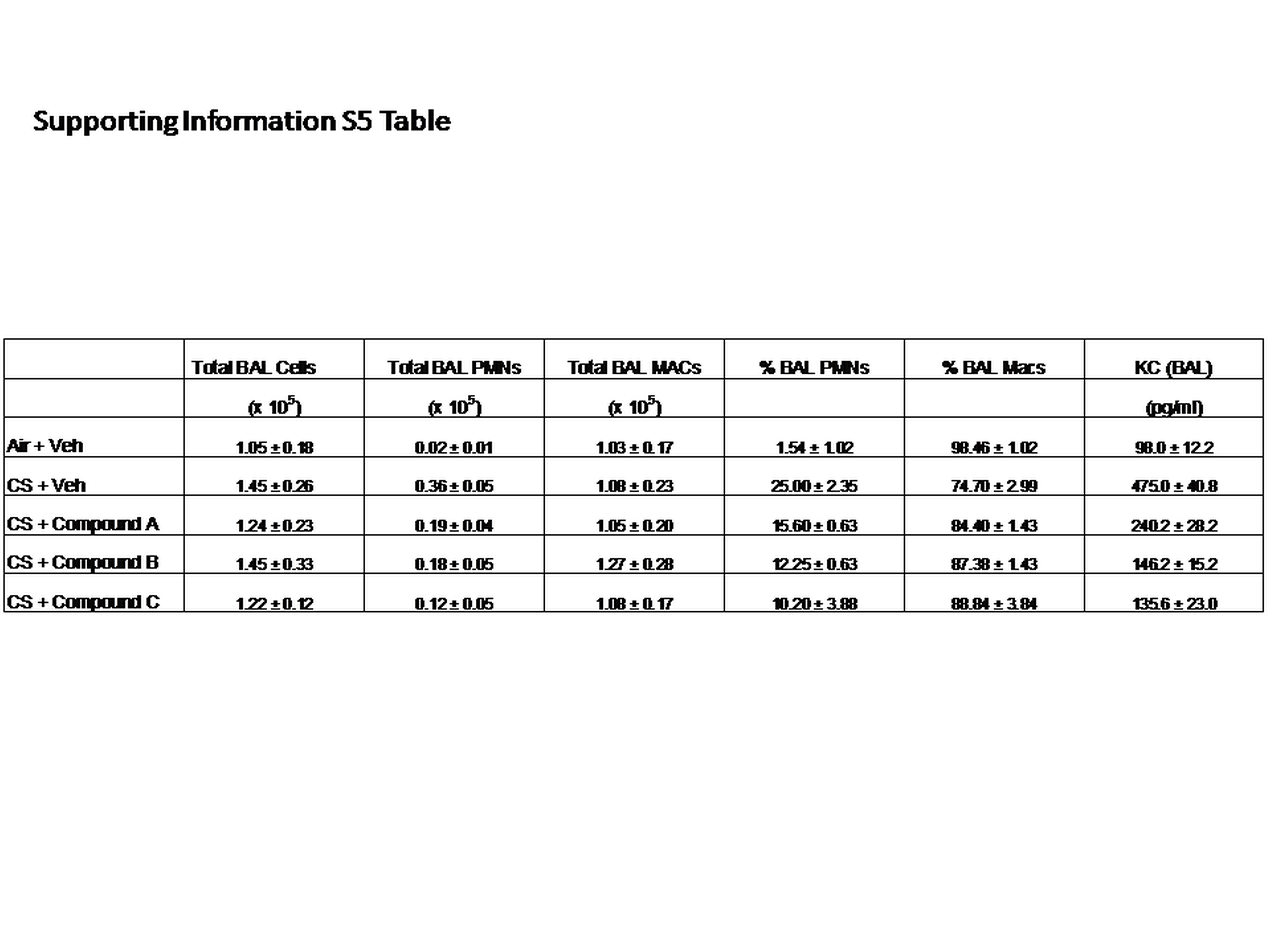

Supplement: S5 Table — The raw cell numbers extrapolated for the graphs shown in Fig 8A and 8B. (TIF) [file pone.0167169.s012.tif]
